# Historical Dynamics of Semi-Humid Evergreen Forests in the Southeast Himalaya Biodiversity Hotspot: A Case Study of the *Quercus franchetii* Complex (Fagaceae)

**DOI:** 10.3389/fpls.2021.774232

**Published:** 2021-11-23

**Authors:** Si-Si Zheng, Xiao-Long Jiang, Qing-Jun Huang, Min Deng

**Affiliations:** ^1^Shanghai Chenshan Botanical Garden, Shanghai, China; ^2^School of Ecological Technique and Engineering, Shanghai Institute of Technology, Shanghai, China; ^3^The Laboratory of Forestry Genetics, Central South University of Forestry and Technology, Changsha, China; ^4^School of Ecology and Environmental Science, Yunnan University, Kunming, China; ^5^Yunnan Key Laboratory of Plant Reproductive Adaptation and Evolutionary Ecology, Yunnan University, Kunming, China

**Keywords:** population genetic structure, ecological niche modeling, *Quercus* section *Ilex*, geoclimatic events, phylogeography

## Abstract

The Oligocene and Miocene are key periods in the formation of the modern topography and flora of East Asian and Indo-China. However, it is unclear how geological and climatic factors contributed to the high endemism and species richness of this region. The *Quercus franchetii* complex is widespread in the southeast Himalaya fringe and northern Indo-China with a long evolutionary history. It provides a unique proxy for studying the diversity pattern of evergreen woody lineages in this region since the Oligocene. In this study, we combined chloroplast (*cp*DNA) sequences, nuclear microsatellite loci (nSSRs), and species distribution modeling (SDM) to investigate the impacts of geological events on genetic diversity of the *Q. franchetii* complex. The results showed that the initial *cp*DNA haplotype divergence was estimated to occur during the middle Oligocene (30.7 Ma), which might have been raised by the tectonic activity at this episode to the Miocene. The nSSR results revealed two major groups of populations, the central Yunnan-Guizhou plateau (YGP) group and the peripheral distribution group when *K* = 2, in responding to the rapid YGP uplift during the late Miocene, which restricted gene flow between the populations in core and marginal areas. SDM analysis indicated that the distribution ranges of the *Q. franchetii* complex expanded northwards after the last glacial maximum, but the core distribution range in YGP was stable. Our results showed that the divergence of *Q. franchetii* complex is rooted in the mid-Oligocene. The early geological events during the Oligocene, and the late Miocene may play key roles to restrict seed-mediated gene flow among regions, but the pollen-mediated gene flow was less impacted. The uplifts of the YGP and the climate since LGM subsequently boosted the divergence of the populations in core and marginal areas.

## Introduction

The late Paleogene (36∼23.3 Ma) is a key period in the formation of the modern topography and flora of Asia (Akhmetiev and Zaporozhets, 2014; Li et al., 2019). During this period, an abrupt climate cooling at the Eocene-Oligocene (E-O) boundary (33.9 Ma) led to the turnover in regional biota and their distribution ranges. Meanwhile, the collisions between the Indian and Eurasian plates greatly changed the topography of Asia (Huchon et al., 1994; Chatterjee et al., 2013; Li S. H. et al., 2017). All these climatic and geological events had profound impacts on the distribution and divergence of the regional biota. However, little is known about how the timing and mechanisms of these ancient geological and climatic events that contributed to the high species diversity and high level of endemism in the southeast Himalayan fringe region.

One prevailing view addressed by numerous scholars is that the rapid uplifts of the Tibetan Plateau-Himalayas (TP) since the Miocene has created new habitats and niches, which have in turn promoted sympatric speciation (Liu et al., 2002; Wang et al., 2007; Meng et al., 2008). Meanwhile the uplift increased the complexity of the regional topography, which efficiently blocked gene flow among the populations, promoting allopatric speciation (Liu et al., 2006; Xu et al., 2010; Li et al., 2013; Qu et al., 2014). However, this view was challenged by Renner (2016) in a review that multiple line of evidences support TP had been 4–5 km high since the mid-Eocene, however, many phylogenetic works in Asia simply attributed the fast speciation between 0.5 and 15 Ma to the fast uplifts of TP, which is either miscited or outdated.

A recent study of Chinese angiosperms by Lu et al. (2018) indicated that the present Chinese flora is young, as a large number of genera did not originate until the Miocene (23 Ma), and their study determined that East China is a “museum” unlike West China, which is a “cradle” of herbaceous species. Likewise, the genome-wide analysis of *Salix brachista* (cushion willow) showed that the TP uplift induced sky island habitats, which increased population differentiation in combination with the Quaternary climate fluctuations, thus boosting *in situ* speciation (Chen et al., 2019). Other phylogenetic studies on relic woody species have also revealed recent divergence events, most dating back to the Miocene, with an intensification in the Pliocene-Pleistocene, regardless of whether these relic lineages had Paleogenic or even more ancient origins, e.g., *Cephalotaxus* (Cephalotaxaceae) (Wang et al., 2014), *Taxus* (Taxaceae) (Gao L. M. et al., 2007; Liu et al., 2013), and *Picea* (Pinaceae) (Shao et al., 2019).

Many study cases have collectively clarified the spatio-temporal diversity pattern of plant lineages in the East Himalayas since the Miocene. Indeed, the uplifts induced environmental heterogeneity and geographic barriers that together triggered the rapid diversification of these subtropical lineages. However, the ancient impacts of the geological events in the Oligocene to early Miocene remain a mystery, as the phylogeographic studies on the Paleogene diverged lineages are quite rare and fossil records in Asia at this epoch are scarce. Nevertheless, there are quite a few Paleogene relic genera also distributed in East Asian and the southwestern Himalayan fringe, e.g., *Ginkgo* (Shen et al., 2005; Gong et al., 2008), *Taxus* (Gao L. M. et al., 2007; Ji et al., 2020), and *Eurycorymbus* (Wang et al., 2009). Phylogeographic studies on those relic species have illustrated that southwestern China, Dabashan, and the Wuyi Mountain regions served as important refugia during time periods with extreme climates (Shen et al., 2005; Gao L. M. et al., 2007; Wang et al., 2009). However, most of these lineages suffered massive extinctions at the geological timescale, with only few extant species with rather restricted distributions remaining. Therefore, investigations of their spatial genetic pattern can only provide limited information about the evolutionary dynamics occurring further back in geological time.

*Quercus franchetii* and *Q*. *lanata* belong to *Quercus* section *Ilex*. The two species are widespread in southwestern China and the southern Himalayas to Northern Indo-China, respectively, at an elevation range of approximately 800–2,600 m (Govaerts and Frodin, 1998; Huang et al., 1999). Their distribution ranges cover the main area of the ancient Red River drainage basin. They are both key regional trees in semi-humid evergreen broad-leaved forests and dry-hot river valleys and have important ecological service and functions (Wangda and Ohsawa, 2006; Liu et al., 2011, 2012). The early derived status of *Q. franchetii* in section *Ilex* at the E-O boundary was inferred by recent phylogenetic studies on oaks (Jiang et al., 2019; Hipp et al., 2020). Our recent phylogenetic study on *Quercus* section *Ilex* supplemented *Q. lanata* for analysis. The result showed that *Q. lanata* and *Q. franchetii* are sister taxa (unpublished data). However, after adding RAD-seq data from more individuals from both species and reconstructing the phylogenetic tree, neither of the two species form a monophyletic clade (unpublished data). Likewise, morphometric measurements showed no difference between *Q. franchetii* and *Q. lanata*, both at the species level as well as among different geographical regions (Zheng, 2021). These results together suggested that the two species are very closely related to each other or indeed represent the same species (hereafter called “*Quercus franchetii* complex”). Data beyond the molecular dating results concur that the *Q. franchetii* complex has a long evolutionary history. Fossils resembling the extant *Q. franchetii* complex have been widely reported along the Tethys/Paratethys Seaway dating to the late Eocene to Pliocene, and they were commonly used as a proxy indicating warm and semi-humid climates (Bouchal et al., 2017; Denk et al., 2017; Guner et al., 2017). Thus, the *Q. franchetii* complex offers a unique opportunity for untangling the timing and possible mechanisms by which geological events since the Oligocene have shaped the high biodiversity and endemism level of the southeastern Himalaya fringe.

**FIGURE 1:**
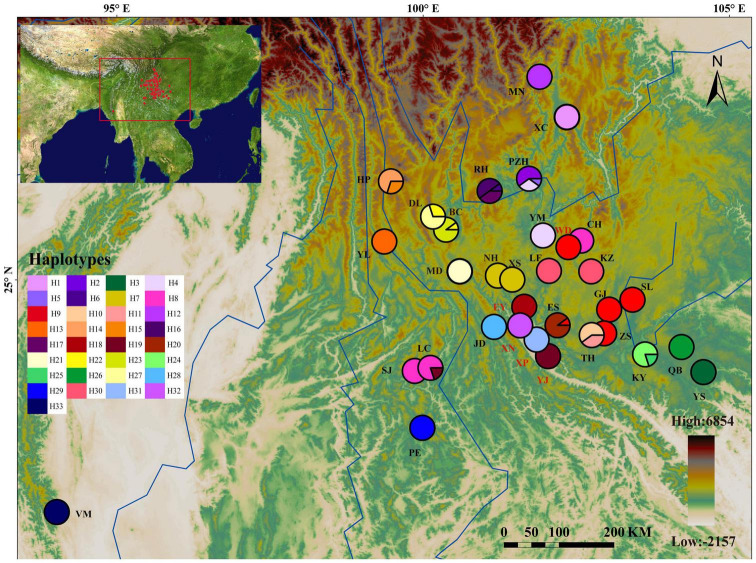
Geographic distribution of 33 *cp*DNA haplotypes detected in the *Quercus franchetii* complex. The colored pie charts representing the frequencies of haplotypes at each sampling site. Haplotype colors corresponding to the charts are shown in the left panel. The color scale representing different elevation gradients are shown in the lower-right panel. The population with less than 10 individuals were marked with red population labels.

In this study, we comprehensively sampled populations of the *Q. franchetii* complex throughout its distribution range (Figure 1). We used *cp*DNA and nSSR markers to scan the populations. By coupling population genetic structure analyses with species distribution modeling (SDM), we aimed to (1) illustrate the spatial genetic structure of the *Q. franchetii* complex, (2) identify the key environmental factors restricting the distribution and diversity pattern of the species complex, and (3) explore the key factors that drove the divergence of the *Q. franchetii* complex. This study provides deep insights into the distribution and evolutionary dynamics of this subtropical woody lineage in the context of global environment change, informing efforts to safeguard this unique forestry ecosystem in the Southeast Himalaya biodiversity hotspot.

## Materials and Methods

### Ethics Statement

Sampling of oaks were granted and supported by National Forestry Bureau of China, Local National Nature Reserves, and Ministry of Environmental Conservation and Forestry, Myanmar.

### Population Sampling

Thirty-three *Q. franchetii* complex populations were sampled from Yunnan and Sichuan, China and Mount Victoria in Chin State, Myanmar. In total, 303 individuals from 33 populations were sampled for this study, covering the major known distribution range of the *Q. franchetii* complex (Table 1). Samples from the same population came from individuals that were separated by at least 50 m. At least 10 trees were sampled from each population, except for populations with very few individuals (WD, EY, YJ, XP, XN), in which case we sampled all the accessible adult trees in those populations. Fresh and healthy mature leaves were collected and put into containers with silica gel to dry them quickly until DNA extractions could be performed. The voucher specimens of the DNA samples were deposited in the herbarium of Shanghai Chenshan Botanical Garden (CSH).

**TABLE 1:** Sampling information, nSSR and *cp*DNA genetic diversity, probabilities of populations belonging to each genetic cluster (*C*_A_, *C*_B_, *C*_C_, *C*_D_) inferred by InStruct analyses, locality habitat suitability, stability obtained from SDMs, and bottleneck effect test for the *Quercus franchetii* complex.

Species	Pop code	Lon	Lat	n	*cp*DNA			SSRs						SDM			Bottleneck
					Haplotypes (no. of individuals)	*h*	π× 10^3^	C_A_	C_B_	C_C_	C_D_	*A*r	*H*e	*N*_Pre_	*N*_LGM_	*N*_stab_	P_W_2t
QF	XC	102.35	27.66	10	H1 (10)	0	0	0.008	0.900	0.083	0.009	3.79	0.532	0.215	0.224	0.991	0.020*
QF	PZH	101.73	26.65	10	H2 (6), H4 (3), H5 (1)	0.6	0.042	0.042	0.928	0.018	0.012	3.83	0.529	0.703	0.457	0.754	0.012*
QF	LF	102.05	25.15	10	H30 (10)	0	0	0.038	0.028	0.932	0.006	3.85	0.557	0.778	0.686	0.909	0.313
QF	YM	101.96	25.72	10	H4 (10)	0	0	0.025	0.937	0.023	0.015	4.17	0.612	0.786	0.655	0.869	0.547
QF	RH	101.08	26.45	10	H6 (1), H16 (5), H17 (4)	0.64	0.028	0.011	0.964	0.009	0.016	3.55	0.519	0.757	0.471	0.714	0.250
QF	NH	101.22	25.07	10	H7 (10)	0	0	0.031	0.048	0.914	0.008	3.96	0.569	0.754	0.627	0.873	1.000
QF	ZXS	101.46	25.01	10	H7 (10)	0	0	0.87	0.033	0.08	0.017	3.87	0.564	0.731	0.598	0.867	0.688
QF	CH	102.58	25.64	10	H8 (10)	0	0	0.141	0.066	0.787	0.006	3.85	0.573	0.688	0.579	0.891	1.000
QF	SJ	99.86	23.51	10	H8 (10)	0	0	0.957	0.017	0.02	0.006	3.23	0.486	0.253	0.391	0.862	0.641
QF	LC	100.12	23.57	10	H8 (8), H19 (2)	0.36	0.011	0.687	0.022	0.286	0.005	3.18	0.518	0.391	0.323	0.932	0.938
QF	GJ	103.04	24.52	10	H9 (10)	0	0	0.978	0.006	0.01	0.006	3.47	0.523	0.760	0.637	0.878	0.383
QF	ZS	102.95	24.13	10	H9 (10)	0	0	0.954	0.008	0.032	0.006	3.38	0.486	0.767	0.658	0.891	0.313
QF	SL	103.42	24.68	10	H9 (10)	0	0	0.073	0.876	0.042	0.009	3.66	0.563	0.627	0.572	0.945	0.313
QF	WD	102.37	25.53	1	H9 (1)	1	0	0.78	0.006	0.209	0.005	−	−	0.684	0.550	0.867	−
QF	ES	102.19	24.26	10	H9 (1), H20 (9)	0.2	0.013	0.932	0.028	0.018	0.023	4.02	0.586	0.707	0.723	0.983	0.055
QF	TH	102.75	24.10	10	H10 (6), H11 (4)	0.53	0.028	0.904	0.022	0.061	0.014	3.46	0.523	0.736	0.635	0.899	0.313
QF	MN	101.90	28.31	10	H12 (10)	0	0	0.009	0.946	0.02	0.025	3.85	0.557	0.509	0.234	0.725	0.945
QF	YL	99.37	25.63	10	H13 (10)	0	0	0.028	0.928	0.04	0.009	3.5	0.536	0.520	0.305	0.785	0.461
QF	HP	99.47	26.61	10	H14 (7), H15 (3)	0.47	0.005	0.007	0.971	0.014	0.009	3.79	0.544	0.386	0.164	0.778	0.250
QF	EY	101.65	24.56	6	H18 (6)	0	0	0.781	0.184	0.028	0.006	3.5	0.568	0.770	0.687	0.917	0.945
QF	YJ	102.04	23.76	7	H19 (7)	0	0	0.841	0.029	0.119	0.011	3.23	0.437	0.638	0.631	0.993	0.020*
QF	MD	100.60	25.14	10	H21 (10)	0	0	0.064	0.024	0.898	0.015	3.39	0.506	0.764	0.643	0.879	0.375
QF	BC	100.37	25.82	10	H22 (1), H23 (9)	0.2	0.026	0.018	0.019	0.958	0.005	3.69	0.530	0.703	0.486	0.783	0.195
QF	DL	100.16	26.03	10	H22 (3), H27 (7)	0.47	0.005	0.808	0.031	0.122	0.039	4.34	0.604	0.653	0.388	0.735	0.074
QF	KY	103.62	23.78	10	H24 (8), H25 (2)	0.36	0.004	0.029	0.772	0.174	0.025	4.01	0.588	0.766	0.667	0.902	0.461
QF	KZ	102.74	25.14	10	H30 (10)	0	0	0.108	0.102	0.765	0.025	3.67	0.540	0.681	0.559	0.879	0.313
QF	XN	101.58	24.26	3	H32 (3)	0	0	0.047	0.021	0.927	0.005	−	−	0.350	0.759	0.591	−
QL	YS	104.58	23.49	10	H3 (10)	0	0	0.925	0.046	0.011	0.018	3.73	0.579	0.465	0.574	0.891	1.000
QL	JD	101.16	24.23	12	H28 (12)	0	0	0.020	0.018	0.942	0.02	3.46	0.549	0.651	0.726	0.924	0.641
QL	QB	104.22	23.91	10	H26 (10)	0	0	0.022	0.94	0.031	0.007	3.95	0.603	0.600	0.565	0.965	0.742
QL	XP	101.85	24.03	3	H31 (3)	0	0	0.868	0.057	0.064	0.011	−	−	0.699	0.713	0.986	−
QL	PE	99.99	22.59	10	H29 (10)	0	0	0.007	0.026	0.014	0.953	3.28	0.522	0.151	0.132	0.981	0.297
QL	VM	94.02	21.21	11	H33 (11)	0	0	0.009	0.009	0.01	0.972	3.7	0.586	0.118	0.074	0.955	0.844

QF, Q. franchetii; QL, Q. lanata; Lon, longitude; Lat, latitude; n, number of individuals investigated in the population; h, haplotype diversity; π, nucleotide diversity; H_e_, expected heterozygosity; A_r_, standardized allelic richness; N_Pre_, present habitat suitability; N_LGM_, last glacial maximum (LGM) habitat suitability; N_stab_, habitat stability since the LGM; P_W_2t, P-values of the TPM model based on the Wilcoxon sign-rank test; (the bold values)*, significant correlation (P < 0.05).

### DNA Extraction, PCR Amplification, and Sequencing

Total genomic DNA was extracted using a modified cetyltrimethyl ammonium bromide (CTAB) protocol (Doyle and Doyle, 1987). Three pairs of *cp*DNA primers, namely *psb*A-*trn*H (Shaw et al., 2005), *trn*T-*trn*L (Taberlet et al., 1991), and *atp*I-*atp*H (Grivet et al., 2001), and eight highly polymorphic nuclear microsatellite loci, specifically nuclear simple sequence repeats (nSSRs), were selected for genotyping on all samples. Primer sequences and PCR amplification conditions are summarized in Supplementary Appendix 1. The *cp*DNA and nSSR amplification conditions followed the methods described by Xu et al. (2015) and An et al. (2016), respectively. PCR products of nSSR markers were 10 times diluted, then mixed with fluorescence size standards at a ratio of 6-FAM: HEX: ROX = 1:1:2, then genotyped by Shanghai Majorbio Bio-pharm Technology Co., Ltd. (Shanghai, China). The PCR products of *cp*DNAs were purified, then biodirectionally sequenced by the same company. All sequences obtained in this study have been deposited in GenBank (see “Data Availability”).

The *cp*DNA sequences were assembled and checked using Sequencher 4.01 (Gene Codes Corp., Ann Arbor, MI, United States). The ClustalW implementation in MEGA X (Kumar et al., 2018) was used for sequence alignment with manual adjustment. All microsatellite loci were checked using GeneMarker^®^ (Hulce et al., 2011) to detect and analyze allele sizes. Null alleles and stutter bands were checked with MicroChecker (Van Oosterhout et al., 2004).

### Genetic Diversity and Structure

Haplotypes and polymorphism statistics for *cp*DNA loci were calculated with DnaSP 6.0 (Rozas et al., 2017). The haplotype geographic distribution was projected onto a map using ArcGIS 10.5.^1^ Total haplotype diversity (*H*_T_), within-population diversity (*H*s), and coefficients of differentiation (*G*_ST_ and *N*_ST_) for *cp*DNA loci were estimated using PERMUT 2.0 (Pons and Petit, 1996). Haplotype diversity (*h*) and nucleotide diversity (π) for *cp*DNA loci were obtained with ARLEQUIN 3.5 (Excoffier and Lischer, 2010). The *cp*DNA haplotype network was constructed using the Median-Joining model in NETWORK 5.0.0.1 (Bandelt et al., 1999). GenAlEx 6.5 (Peakall and Smouse, 2012) was used to calculate the genetic diversity index of microsatellite data, including expected heterozygosity (*H*_e_), observed heterozygosity (*H*_o_), number of alleles (*N*_A_), and effective number of alleles (*N*_E_). Allelic richness (*A*_r_) of nSSRs was determined using HP-RARE (Kalinowski, 2005).

The genetic differentiation values (*F*_ST_) based on *cp*DNA and nSSR data were estimated using ARLEQUIN 3.5, respectively (Excoffier and Lischer, 2010). The Genetic Landscape GIS toolbox (Vandergast et al., 2011) in ArcGIS 10.5 was used to generate a geographical landscape map based on both genetic diversity (*A*_r_) for *cp*DNA loci and genetic divergence (*F*_ST_) based on *cp*DNA and nSSR data according to inverse distance weighted interpolation. For *cp*DNA and nSSR data, analysis of molecular variance (AMOVA) was performed to assess the genetic variation among populations and within populations. The Mantel test was also performed based on the genetic structure and geographic distance matrix with 1,000 random permutations to evaluate their relationship and test isolation by distance (IBD). For nSSR data, Principal coordinate analysis (PcoA) based on genetic distance was performed using GenAlex 6.5 (Peakall and Smouse, 2012) to assess differences among individuals or groups. Barrier (Manni et al., 2004) was used to set up geographic barriers according to sample locations to detect the existence of genetic barriers among populations.

We used GenePop v 4.2 (Rousset, 2008) to test departures from Hardy-Weinberg equilibrium (HWE) for each of the eight nSSR loci. As the nSSR loci significantly deviated from HWE (see Results section “Genetic Diversity and Structure”), Bayesian assignment probability methods using the programs InStruct (Gao H. et al., 2007) and STRUCTURE 2.3.4 (Pritchard et al., 2000) were both used to infer the population genetic structure. The number of clusters (*K*) was varied from 1 to 10, with 10 replicates at each value. Each run consisted of a burn-in length of 25,000 iterations with a run length of 500,000 MCMC (Markov chain Monte Carlo) iterations. The optimal *K* (number of clusters) was determined using the △*K* method (Evanno et al., 2005). CLUMPP (Jakobsson and Rosenberg, 2007) was used to align 10 runs of InStruct with the optimum *K* using a greedy algorithm.

Among the three models in the Bottleneck program (Luikart et al., 1998), the “Two-phase mutation model” (TPM) is the most suitable for microsatellite loci, which was thus selected to test whether the populations of the *Q. franchetii* complex had experienced a bottleneck. We used “Wilcoxon sign-rank test” method for a significance test.

### Divergence Time Estimation

The divergence time dating for *cp*DNA haplotypes of the *Q. franchetii* complex was estimated using a Bayesian approach as implemented in BEAST V2.4 (Suchard et al., 2018). *Quercus glauca* (*Quercus* section *Cyclobalanopsis*) was chosen as the outgroup to root the tree. An uncorrelated lognormal relaxed clock was applied with the K81uf + I substitution model, which was selected based on the Akaike information criterion (AIC) in Modeltest 3.7 (Posada and Crandall, 1998). The earliest conclusive leaf fossils of *Quercus* section *Ilex* were discovered in Tibet, dated to the late Eocene (ca. 34 Ma) (Su et al., 2019) was set as the minimum age to constrain the stem of the haplotype tree of the *Q. franchetii* complex with a lognormal distribution and a median of 37.8 Ma (95% HPD: 34.02–56.6 Ma). The MCMC chains were run for 100 million generations with a sampling frequency of once every 10,000 generations. Convergence was assessed using Tracer v1.7^2^ (Rambaut et al., 2018), and the effective sample sizes for all parameters were calculated. The resulting tree and log files from the two replicate runs were combined with LogCombiner v1.8. Then, we used TreeAnnotator v. 1.8^3^ to generate the maximum clade credibility (MCC) tree after discarding the first 20% of the trees as burn-in. The results were visualized using FigTree v1.4.3.^4^

### Population Demographic History and Ancestral Area Reconstruction Analyses

Pairwise mismatch distribution analysis for *cp*DNA loci, Tajima’s (1989) *D* and Fu’s (1997) *F*s of neutrality tests were performed to detect possible demographic expansions of the *Quercus franchetii* complex using ARLEQUIN 3.5 (Excoffier and Lischer, 2010).

**FIGURE 2:**
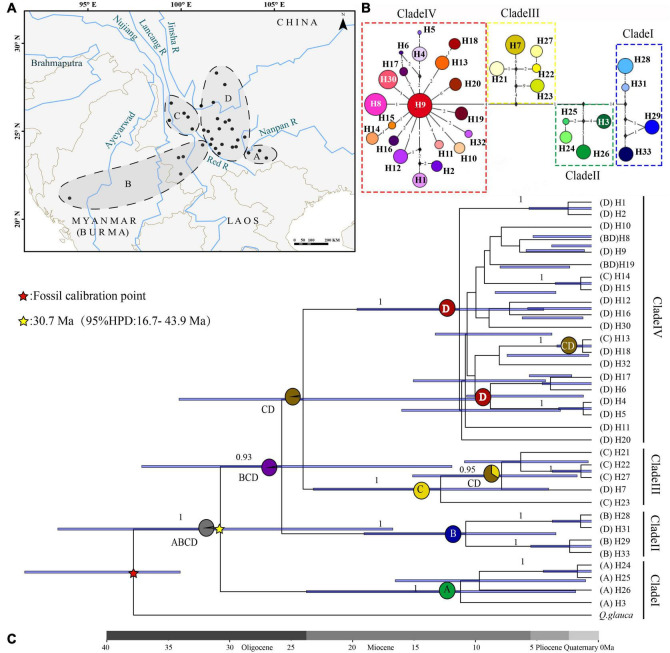
Phylogenetic analysis and ancestral distribution area reconstruction of the *Quercus franchetii* complex. **(A)** Ancestral distribution area of the *Q. franchetii* complex. A, Nanpan River region (NPR); B, southwestern Red River (RR); C, Hengduan Mountains (HDM); D, Yunnan–Guizhou Plateau (YGP). **(B)** Haplotypes of the *cp*DNA network of the *Quercus franchetii* complex. The colors in the pie charts represent different *cp*DNA haplotypes. Numbers on the branches indicate the number of substitutions. Black diamonds indicate unsampled or extinct ancestral haplotypes. **(C)** BEAST-derived chronogram of the *Q. franchetii* complex. The blue bar length represents the 95% HPD of the species divergence time, and circles at nodes represent the distribution range. Values below branches represent the posterior probability.

Ancestral range reconstruction was analyzed using statistical dispersal-vicariance (S-DIVA) analysis as implemented in RASP (Yu et al., 2015) and DEC models (Ree et al., 2005; Ree and Smith, 2008). Four areas were delimited for ancestral area reconstruction based on the geological characteristics and biogeographical division of southwestern China, including (A) the Nanpan River region (NPR); (B) the southwestern Red River (including southern Himalayas to Red River) (RR); (C) the Hengduan Mountains (HDM), and (D) the Yunnan-Guizhou Plateau (YGP) (Figure 2A).

### Species Distribution Modeling

We used MaxEnt 3.4 (Phillips et al., 2006) to simulate the potential distribution range of the *Q. franchetii* complex (including *Q. franchetii* and *Q. lanata*) under the past, current, and projected future climate scenarios based on the maximum entropy model (Phillips et al., 2004). In total, 145 accurate occurrence points were collected from the Global Biodiversity Information Facility (GBIF),^5^ Chinese Digital Herbarium (CVH),^6^ and the field collection records of our research team. Each voucher specimen in the distribution record was inspected and checked carefully. Nineteen bioclimatic variables with a 2.5-arc-min resolution for the present (1950–2000), the last glacial maximum (LGM) (CCSM) period, and the future (2060–2080, RCP2.6; RCP4.5; RCP6.0; RCP8.5) were downloaded from WorldClim 2.^7^ The nine environmental factors (bio1, Annual Mean Temperature; bio4, Temperature Seasonality; bio6, Min Temperature of Coldest Month; bio7, Temperature Annual Range; bio10, Mean Temperature of Warmest Quarter; bio11, Mean Temperature of Coldest Quarter; bio12, Annual Precipitation; bio13, Precipitation of Wettest Month; bio17, Precipitation of Driest Quarter) were selected after eliminating climatic variables such that none that were include were highly correlated (i.e., with correlation coefficients greater than 0.8) using the Dismo package in R.^8^ We also chose CHELSA climate data,^9^ which had a higher prediction power in mountain regions (specifically the Himalayas), to simulate the present distribution (Bobrowski and Schickhoff, 2017; Karger et al., 2017).

In order to improve the prediction accuracy of the model, it was necessary to optimize the model in MaxEnt 3.4 (Phillips et al., 2006) by setting the β multiplier and environmental characteristic parameters. The MaxEnt model captures five features: linear (L), quadratic (Q), hinge (H), product (P), and threshold (T). In this research, we used seven feature combinations (auto, L, H, LQ, LPQ, LQH, LQHP) and set the regularization multiplier from 0.5 to 10 with increments of 0.5. Then, we used ENMTools (Warren et al., 2010) to calculate the lambdas file for the maxent result, and selected the model with the smallest AIC value as the optimal model parameter for further analyses. The occurrence points of *Q. franchetii* and *Q. lanata* were randomly divided into 75 and 25% of the data for training and testing, respectively. The area under the curve (AUC) was used to evaluate the model’s accuracy. AUC values ranged from 0.5 to 1, where the higher AUC value indicated a better prediction. The maximum training sensitivity plus specificity thresholds for the presence or absence of species was used to draw a species distribution map in ArcGIS 10.5. In addition, we classified three categories equally between this threshold and 1, corresponding to low, medium, and high fitness areas, respectively. To compare the changes in species distribution across different periods, three indicators were calculated: locality habitat stability (*N*_Stab_), habitat distribution area ratio (*N*_a_), and habitat expansion extent (*N*_*e*_). These values were calculated using the following formulas: *N*_Stab_ = 1- | *N*_Pre_ - *N*_LGM_ |, where *N*_Pre_ and *N*_LGM_ are the habitat suitability of the present and LGM distribution area; *N*_*a*_ = (present distribution areas)/(LGM or future distribution areas): a value close to 1 indicates a stable distribution of the species, while a value much higher or lower than 1 indicates that the distribution area of the species has expanded or contracted from the LGM to the present; *N*_*e*_ = [1 - (Distribution area overlapping between the LGM and present or the future and present/present distributions area)] × 100% represents the percentage of the distribution that has expanded from the LGM to the present.

The potential dispersal routes of the *Q. franchetii* complex in the past and present periods were inferred based on the least-cost path analysis method using SDM toolbox 2.0 (Brown, 2014) in ArcGIS 10.5. The specific steps are listed below: Firstly, we generated a resistance layer by inverting the SDMs (1-SDM). The resistance layer was used to create a cost distance raster for each sample locality. The corridor layers were built between two locations that only share haplotypes based on the cost distance raster. We used the categorical the least cost path (LCP) approach to better describe the habitat heterogeneity and its role in the dispersal. The value of each corridor layer was divided into low, medium, and high, and then these three intervals were re-divided into new values 5, 2, and 1. Finally, we reclassified all the corridor layers, summarized and standardized them from 0 to 1, and determined the dispersal corridors of the *Q. franchetii* complex.

### Detection of Correlations Between Genetic Diversity and Climatic Factors

The linear model in R 3.5^10^ was used to estimate the correlation of genetic diversity indexes (*A*_r_ and *H*_e_) and genetic structure (cluster A from the Bayesian clustering, C_A_) with habitat and geographic factors. Five variables—population longitude and latitude, habitat suitability for the present (*N*_Pre_) and the LGM (*N*_LGM_), and habitat stability (*N*_Stab_) were used as explanatory covariates.

## Results

### Genetic Diversity and Structure

**FIGURE 3:**
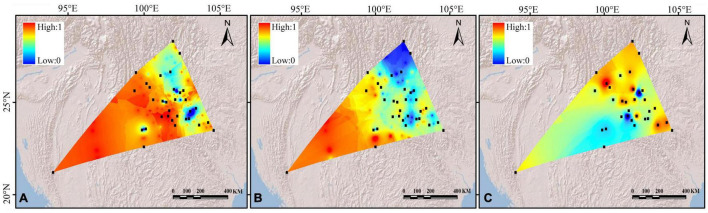
Spatial interpolation of genetic differentiation (*F*st) **(A)** *cp*DNAs, **(B)** nSSRs, and **(C)** genetic diversity of the *Quercus franchetii* complex based on *cp*DNAs. The color ranges from blue to red, representing the genetic differentiation or genetic diversity values from low to high.

The *psb*A-*trn*H, *atp*I-*atp*H, and *trn*T-*trn*L sequence alignments were 482–520, 899–1030, and 796–838 bp in length, respectively. The combined length of the three aligned chloroplast fragments was 2,319 bp, with 94 polymorphisms across a total of 33 haplotypes among the 303 individuals analyzed. The *cp*DNA haplotype diversity was *h* = 0.956 and nucleotide diversity was π = 0.00536. The nucleotide diversity and haplotype diversity within the populations were 0–0.04184 and 0–0.644, respectively (Table 1 and Figure 1). The total diversity (*H*_T_ = 0.982) was much higher than the average within-population diversity (*H*_S_ = 0.123). The genetic diversity map showed that the NPR and HDM populations had high genetic diversity (Figure 3C). *N*_ST_ (0.959) was significantly greater than *G*_ST_ (0.874) (*P* < 0.05), which indicated that a clear phylogeographic structure existed among the *Q. franchetii* complex populations. The network analysis resolved four distinct *cp*DNA haplotype clades in the *Q. franchetii* complex, which was consistent with the haplotype structure determined by BEAST. The haplotypes in Clade IV exhibited a star-like structure. The most common haplotype, H9, had the widest distribution, mainly within the central YGP (Figure 2B).

Among the 303 individuals genotyped, a total of 115 alleles were identified using eight pairs of microsatellite primers. All eight microsatellite loci were highly polymorphic, with allele numbers varying from 8 to 18 per locus. The genetic diversity indexes *N*_A_, *N*_E_, *H*_e_, and *H*_o_ were 4.182 (SE = 0.103), 2.578 (SE = 0.066), 0.537 (SE = 0.013), and 0.536 (SE = 0.016), respectively, while allele richness (*A*_r_) was 3.18–4.34.

**FIGURE 4:**
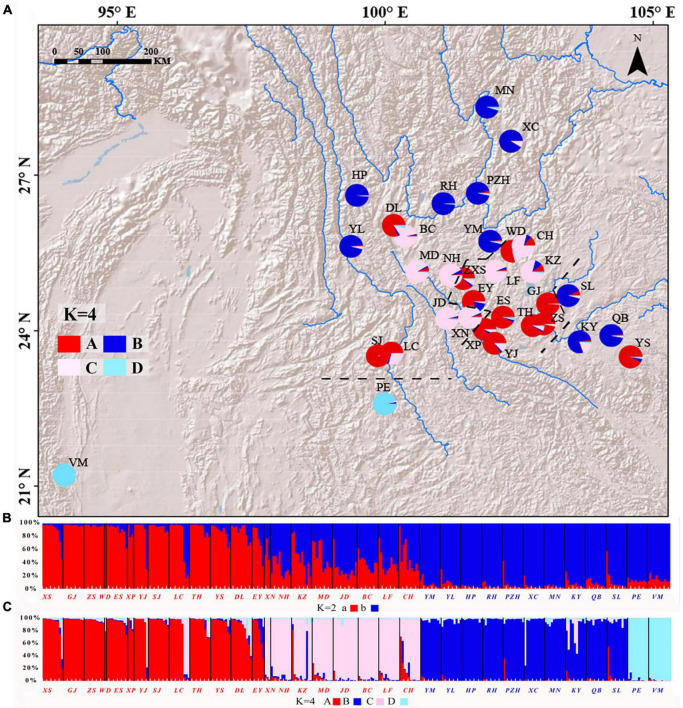
**(A)** Geographic distribution of the *Quercus franchetii* complex according to STRUCTURE grouping analyses. STRUCTURE cluster analysis diagram when **(B)** *K* = 2 and **(C)** *K* = 4. The colors in the pie charts represent different groupings, and the black dotted line indicates the inferred geographic isolation based on Barrier.

The AMOVA on *cp*DNA sequence data revealed greater genetic variation among populations (87%; *F*_ST_ = 0.87) than within populations (13%). In contrast, the nSSRs showed substantial genetic differences within populations (63%; *F*_ST_ = 0.369, Table 2). Pairwise *F*_ST_ values calculated based on chloroplast and SSR data were shown in Supplementary Appendix 2. In order to more visually observe genetic differentiation, *F*_ST_ values were projected onto a map and a genetic divergence map was made. The genetic divergence map showed that the population in southwestern Yunnan had a relatively high genetic divergence, while the divergence of the populations from the northeast was comparatively lower (Figures 3A,B). The Mantel tests of *cp*DNA data (*r* = 0.052, *P* = 0.001) and microsatellite data (*r* = 0.413, *P* = 0.001) both revealed significant correlations between genetic and geographic distances, but the correlations among chloroplast markers were weaker (Supplementary Appendix 3). Barriers inferred from nuclear genes showed a barrier between the YGP and HDM regions (Figure 4). As well as, distinct geographical isolation had been detected between the populations in southwestern Yunnan and NPR (Figure 4). Based on the TPM model, three populations were determined to have experienced bottleneck (PZH, XC, YJ; Table 1).

**TABLE 2:** Molecular variance analysis of the *Quercus franchetii* complex.

	Source of variation	d.f.	Sum of squares	Variance components	Percentage of variation%
***cp*DNA**	Among populations	31	126.684	0.427	87%
	Within populations	270	17.200	0.064	13%
	Total	301	143.884	0.491	100%
	Among populations	31	1014.597	2.939	37%
**SSR**	Within populations	270	1356.910	5.026	63%
	Total	301	2371.507	7.965	100%

A total of three loci of nSSRs conformed to HWE, and five loci deviated from HWE (*P* < 0.05, Supplementary Appendix 1). In the Bayesian clustering analysis, the optimal *K*-value for STRUCTURE was *K* = 4 (Supplementary Appendix 4). However, the optimal *K*-value selected by InStruct was 2, and the second highest peak occurred for *K* = 4 (Supplementary Appendix 4). Thus, we performed cluster analysis on the *Q. franchetii* complex using *K* = 2 and *K* = 4, respectively for both STRUCTURE and InStruct. Following the InStruct runs with *K* = 2, cluster a was mainly composed of the YGP group, with some populations in the HDM (DL, BC, MD), and southwestern Yunnan (LC, SJ) groups. Cluster b was composed of 12 populations peripheral to those of cluster a. When *K* = 4, the YGP group was further divided into subgroups, and the populations from RR (PE, VM) separated from the rest of cluster b to form a new subgroup (Figure 4). The STRUCTURE results were identical to the results obtained from STRUCTURE (Supplementary Appendix 5). The principal coordinate analysis (PcoA) of the clustering results found that 33 natural populations can be divided into four regions, which is mostly consistent with the results obtained using InStruct. The first and second principal components explained 16.24 and 6.91% of the genetic variation, respectively (Supplementary Appendix 6).

### Divergence Time Estimation

The crown age of *cp*DNA haplotypes in the *Q. franchetii* complex was dated to the late Oligocene (30.7 Ma, 95% highest posterior density, HPD = 16.7–43.9 Ma), and Clade I (NPR) was the earliest derived. The haplotypes of the southwestern lineage (Clade II) began to diversify around 25.7 Ma (95% HPD = 11.9–37.2 Ma). The divergence of Clades III (HDM region) and IV (YGP region) was dated to ca. 24 Ma (95% HPD = 9.95–34.13 Ma), with subsequent rapid divergence of the haplotypes during the late Miocene (Figure 2).

### Demographic History and Ancestral Area Reconstruction

#### Demographic History

Neutrality tests (Tajima’s *D* = −0.50558, *P* = 0.325; Fu’s *F*s = 2.77576, *P* = 0.789) failed to identify population expansion. The mismatch distribution for *cp*DNA showed a multimodal distribution, consistent with a stable population size (Supplementary Appendix 7).

#### Ancestral Area Reconstruction

The ancestral distribution range reconstruction of the S-DIVA and DEC models both showed that the *Q. franchetii* complex had a wide distribution of ancestral populations. The S-DIVA model (95% HPD = 99%) provided a higher confidence estimate than did the DEC model (95% HPD = 45.25%), and its results indicated that the ancestral distribution area of the *Q. franchetii* complex was widespread in southwestern Yunnan and the Southern Himalayas (including A, B, C, and D). Then followed three vicariance events, which led to the divergences of the NPR lineage (Clade I) during the Oligocene (95% HPD = 99%) and the RR lineage (Clade II) during the late Oligocene episode (95% HPD = 98.97%), with an increase in the divergence of the HDM lineage (Clade III) and YGP lineage (Clade IV) during the early Middle Miocene (Figure 2). The DEC results are shown in Supplementary Appendix 8.

### Ecological Niche Modeling

**FIGURE 5:**
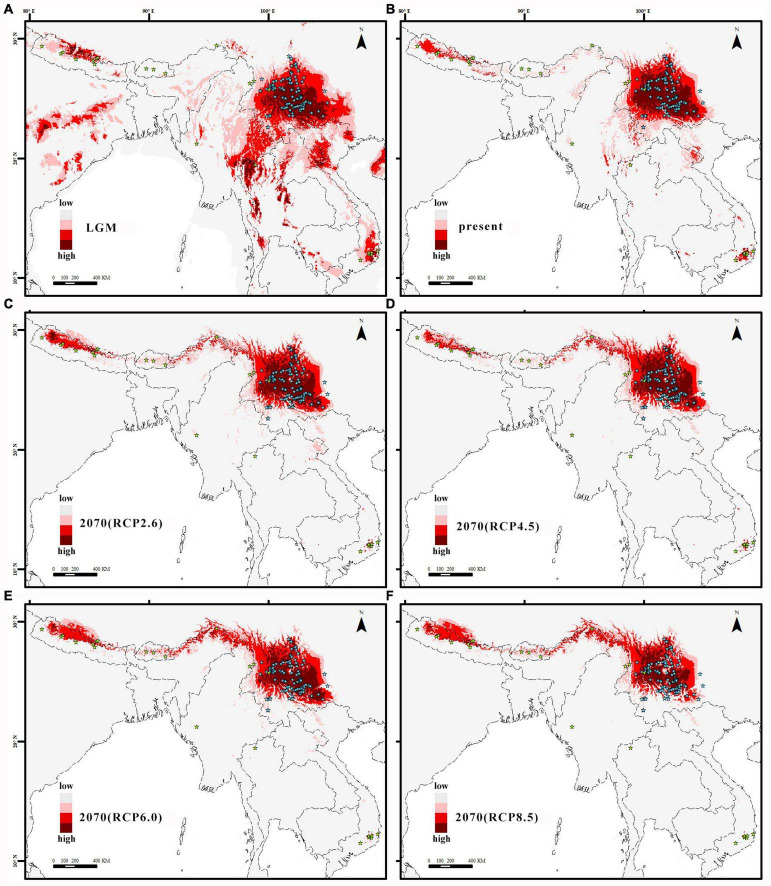
**(A)** the last glacial maximum (LGM), **(B)** the present, **(C)** in 2070 under the RCP2.6 scenario, **(D)** in 2070 under the RCP4.5 scenario, **(E)** in 2070 under the RCP6.0 scenario, and **(F)** in 2070 under the RCP8.5 scenario. The color from pink to red represents the fitness zone, from low to high respectively.

Ecological niche modeling of the *Q. franchetii* complex in Maxent using WorldClim climate data revealed a high performance score (AUC = 0.9679–0.9719, standard deviation = 0.0157). Annual Mean Temperature (bio1) was the greatest contributor (36.46%, standard deviation = 1.3), followed by Temperature Seasonality (bio4) (34.7%, standard deviation = 1.3) and Annual Precipitation (bio12) (7.72%, standard deviation = 1.52) in identifying the areas of occurrence for *Q. franchetii* complex populations. The maximum sensitivity plus specificity value 0.13 was used as the species absence/presence threshold. The current distribution of the *Q. franchetii* complex was similar to the predicted distribution, except for quite a few occurrence sites in southwestern and southeastern Yunnan and Southern Himalayas that were located in predicted unsuitable areas (Figure 5B). The potential distribution range retreated to the south during the LGM (Figure 5A). The total distribution area of the LGM was greater than that of the present (*N_*a*_* = 0.42, *N_*e*_* = 21%). In the future period, the higher the RCP value was, the more obvious of an expansion in the suitable area to the northwest was detected. The ratios of the present range to the future range under RCP2.6, RCP4.5, RCP6.0, and RCP8.5 were 1.07, 1.15, 1.04, and 1.18, respectively. Accordingly, the highly suitable habitat would be reduced by 7.2, 7.2, 16.5, and 40.1%, respectively (Figures 5C–F). The predicted present distribution of *Q. franchetii* complex using CHELSA climate data was similar to that from WorldClim climate data (Supplementary Appendix 9). The suitable distribution area in the current period from the two data sources was largely overlapped (0.94). In order to better compare the distribution dynamics with other oak, of which WorldClim database were generally used for distribution simulation, we selected the results of WorldClim for the subsequent analyses.

**FIGURE 6:**
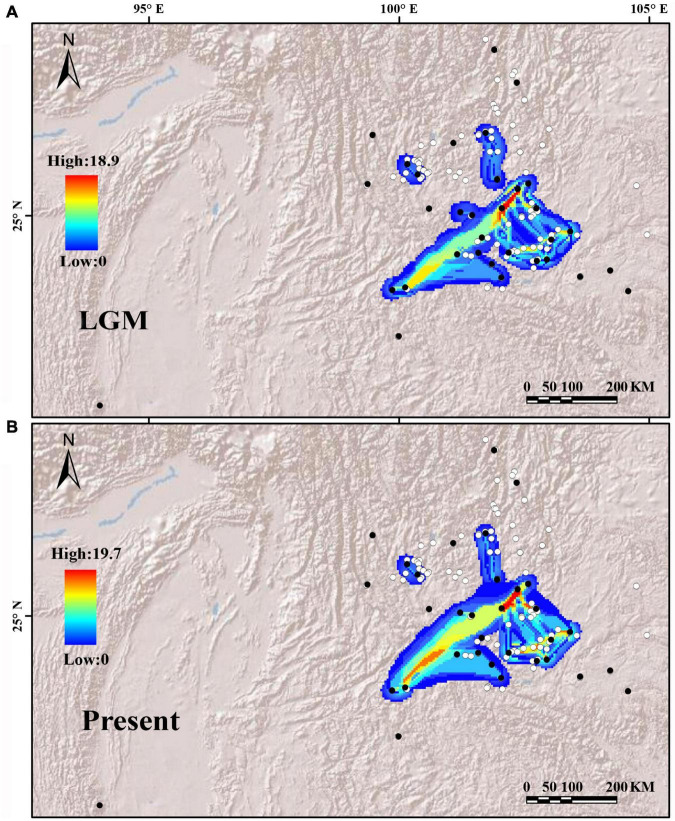
Potential dispersal corridors of the *Quercus franchetii* complex during **(A)** the last glacial maximum (LGM) and **(B)** the present. Both white and black dots are the occurrence points of the *Q. franchetii* complex, with the black dots indicating sampling sites used in this study and white points indicating occurrence sites obtained from herbarium records. Colors in **(A,B)** from blue to red represent the potential of species dispersal corridors from low to high.

Putative dispersal corridors in the two periods were visualized based on *cp*DNA haplotype diversity (Figure 6). The dispersal corridors during the two periods are consistent, and both showed that the YGP area had a higher dispersal ratio, but the populations of the peripheral areas were rather isolated.

### Correlation Between Genetic Diversity and Climatic Factors

The correlation analysis indicated that only the *N*_LGM_ was significantly correlated with GenPCoA1 (*P* < 0.05). *A*_r_ was associated with both longitude and latitude (*P* < 0.05). Cluster A (*C*_A_) and GenPCoA1 were both associated with latitude (*P* < 0.05). However, *H*_e_ was not associated with *N*_Pre_, *N*_stabLGM_, *N*_LGM_, latitude, nor longitude (*P* > 0.05) (Table 3).

**TABLE 3:** Correlation between genetic diversity, habitat and geographical factors of *Quercus franchetii* complex.

	*A*r				*H*e				C_*A*_				GenPCoA1			
	Estimate	Se*^a^*	t	*p*	Estimate	Se*^a^*	t	*p*	Estimate	Sea	t	*p*	Estimate	Sea	t	*p*
(Intercept)	−7.44	4.00	−1.86	0.07	−0.70	0.59	−1.18	0.25	4.03	1.50	2.68	0.01*	14.42	6.35	2.27	0.03*
*N*_Pre_	−	−	−	−	−	−	−	−	−	−	−	−	−	−	−	−
*N*_stadLGM_	−	−	−	−	−	−	−	−	−	−	−	−	−	−	−	−
*N*_LGM_	−	−	−	−	−	−	−	−	−	−	−	−	1.31	0.51	−2.98	0.01*
longitude	0.08	0.04	2.16	0.04*	0.01	0.01	1.89	0.07	−	−	−	−	−0.11	0.06	−1.75	0.09
latitude	0.13	0.04	3.20	0.004 *	0.01	0.01	1.50	0.15	−0.15	0.06	−2.44	0.02*	−0.17	0.06	−2.98	0.01*

*Significant correlation (P < 0.05), Se^a^, standard error.

## Discussion

### Genetic Diversity Pattern of the *Quercus franchetii* Complex

Our study revealed a high genetic diversity estimate for the *Q. franchetii* complex (*H*_T_, 0.982; *A*_r_, 3.18–4.34), which is similar to the genetic diversity of other evergreen oaks in southwestern China, e.g., *Q. schottkyana* (*H*_T_, 0.828; *A*_r_, 4.83–7.78; Jiang et al., 2016), *Q. kerrii* (*H*_T_, 0.71; *A*_r_, 2.27–3.20; Jiang et al., 2018), *Q. delavayi* (*H*_T_, 0.907; *A*_r_, 3.750–5.237; Xu et al., 2020), and the *Q. cocciferoides* complex (*H*_T_, 0.904; Liu, 2019). Comparatively, the genetic diversity for deciduous oaks seems lower, e.g., in the eight European white oaks (H_T_, 0.635–0.847) (Petit et al., 2002), *Quercus variabilis* (*H*_T_, 0.888, in 50 populations in East Asia) (Chen et al., 2012), *Q. mongolica* var. *crispula* (*H*_T_, 0.827, in Japan) (Okaura et al., 2007), *Q. acutissima* (*H*_T_, 0.791, in Southeast China) (Zhang et al., 2015). The deciduous species are mainly Miocene-Pliocene derived young lineages, but the divergence time of the evergreen oaks are much older that dated to the Eocene-Miocene (Hipp et al., 2020). It is easy to understand that oak taxa with long evolutionary histories and wide distribution can accumulate higher levels of genetic diversity than those “young” species. Additionally, the high environmental heterogeneity of southwestern China can buffer the climate extremes of the Quaternary episode (Zhang et al., 2010; Kai and Jiang, 2014; Jiang et al., 2018). As a result, the habitats of southwestern China have had long-term stability without significant regional extinction events or distribution range shifts to allow the genetic diversity of the species can be maintained. All these factors contributed to the high genetic diversity level found in evergreen oaks in southwestern China.

Comparing the genetic structure of the sympatric close related species can better illustrate the factors determining genetic diversity pattern. Notably, there was significant genetic differentiation among populations in the *Q. franchetii* complex, with *F*_ST_ estimates for *cp*DNA and nSSRs of 0.87 and 0.369, respectively, which is very similar to that found in *Q. delavayi*, as the *cp*DNA of the both species show significant phylogeographic structure, IBD pattern, and high differentiation among the populations (Xu et al., 2020). However, in two other sympatric/parapatric oaks, *Q. kerrii* (Jiang et al., 2018) and *Q. schottkyana* (Jiang et al., 2016), only low differentiation among the populations without phylogenetic structure were found (Table 4). The biological traits restrict gene flow and its efficiency, no doubt, can greatly impact the population genetic structure (Petit et al., 2003; Cavender-Bares et al., 2015; Xu et al., 2020). Pollen and seed mediated gene flow among the populations is different, as they have different dispersal efficiencies when barriers exist (e.g., rivers and high mountains). Generally, the seed-mediated gene flow among populations is more restricted in the species with instant germination seeds comparing to those species that seed with a period of dormancy. Consequently, the typical recalcitrant seed species shows significant phylogeographic structure in *cp*DNA makers. Vice versa, as pollen of oaks can disperse long distance, population differentiation revealed by biparental markers was much lower than that in maternal makers (Xu et al., 2020). However, the genetic structure of *Q. franchetii* seems not only determine by seed/pollen mediated gene flow efficiency. Although *Q. franchetii* has temporary seed dormancy (2–4 months; Xia et al., 2012), its seed size and tannin content similar to those of *Q. schottkyana*, but its population genetic structure is similar to that of *Q. delavayi—*a typical recalcitrant small seeds species. Thus, factors beyond seed germination schedule, seed size, and dispersal abilities also played important roles in shaping the population structure of these oaks in southwestern China, e.g., their ancestor distribution range and evolutionary history, etc.

**TABLE 4:** Comparisons of the genetic diversity, genetic structure, and demographic dynamics of *Quercus franchetii* complex with *Q. schottkyana*, *Q. kerrii*, *Q. schottkyana*, and *Q. cocciferoides* complex.

Taxa	Genetic diversity		Genetic structure						Demographic change			IBD	
	*H*_T_ (se)	*H*_S_ (se)	*G*_ST_ (se)	*N*_ST_ (se)	Phylo structure	Network structure	*F*_ST_ (b)	*F*_ST_ (m)	*Na*	Mismatch	Neutral test	*R* (m)	*R* (b)
*Q. franchetii* complex	0.982 (0.01)	0.123 (0.04)	0.874 (0.04)	0.959* (0.02)	No	Star-like	0.369	0.87	0.42	No expansion	No expansion	0.052*	0.413*
*Q. delavayi*	0.907 (0.03)^a^	0.197 (0.05)^a^	0.782 (0.05)^a^	0.912* (0.03)^a^	Yes^a^	Geographic structure^a^	0.063^a^	0.938^a^	1.14^a^	No expansion^a^	No expansion^a^	0.587*^a^	0.365*^a^
*Q. kerrii*	0.71 (0.06)^b^	0.05 (0.02)^b^	0.93 (0.03)^b^	0.92 (0.04)^b^	No^b^	Star-like^b^	0.066^a^	0.894^a^	1.27^b^	Expansion^a^	No expansion^b^	−	−
*Q. schottkyana*	0.828 (0.06)^a^	0.341 (0.06)^a^	0.588 (0.07)^a^	0.615 (0.11)^a^	No^a^	Star-like^c^	0.075^a^	0.665^a^	1.03^c^	No expansion^c^	No expansion^c^	−	−
*Q. cocciferoides* Complex	0.904^d^	0.140^d^	0.845^d^	0.860^d^	Yes^d^	Geographic structure^d^	0.134^d^	0.946^d^	1.29^d^	No expansion^d^	No expansion^d^	0.312^d^	0.886^d^

H_T_, total haplotype diversity; H_S_, within population diversity; G_ST_, coefficient of genetic variation over all populations; N_ST_, coefficient of genetic variation influenced by both haplotype frequencies and genetic distances between haplotypes; Phylo structure, phylogeographic structure; F_ST_(b), population differentiation for nuclear; F_ST_(m), population differentiation for maternally inherited; Na, habitat distribution area ratio; R(m), correlation of two matrices for maternally inherited; R(b), correlation of two matrices for nuclear; *N_ST_ differs from G_ST_ at P < 0.05.

*^a^Xu et al. (2020); ^b^Jiang et al. (2018); ^c^Jiang et al. (2016); ^d^Articles to be published.*

Contemporary and historical factors shaped the genetic structure of organisms (Van Oppen et al., 2011; Hernawan et al., 2017; Li J. J. et al., 2017). Geological and climatic factors have been shown to influence the evolutionary histories of taxa and shape their genetic structures (Feng et al., 2014; Xing et al., 2014; Lu et al., 2018; Chen et al., 2020). Thus, the genetic structures of ancient lineages with long evolutionary histories and wide distribution range can essentially record more ancient geological events than in those of young lineages. The evergreen oak lineages in YGP and southwestern China, e.g., *Q. schottkayana* at 6.37 Ma (Jiang et al., 2016), *Q. kerrii* at 6–7 Ma (Jiang et al., 2018), and *Q. cocciferoides* at ca. 5 Ma (Jiang et al., 2019; Liu, 2019) are later derived “young” (originated at the late Neogene) and they generally show no (or very limited) IBD pattern among the populations. In contrasts, the early derived species *Q. delavayi* with crown node age at 10.92 Ma, and the *Q. franchetii* complex with crown node age at 30.7 Ma (95% HPD 16.7–43.9 Ma) had similar genetic structures and distinct IBD pattern. The different genetic structures detected in the “young” and “old” species might reflect the outcomes of past geological events on the biota at the different epochs, as the ancient geological events may imprint a genetic structure pattern in an “old” and widespread lineages, but not in those of the “young” lineages. However, our hypothesis requires further investigation, as the molecular markers we used in previous phylogeographical studies on oaks of southwestern China are not all the same. Regardless, these markers are informative to reveal the spatial genetic structures of these oaks, but it contains potential bias when comparing across the species to reveal the underlying mechanisms that shaped the genetic structures of these oaks. Further studies using universal high-throughput markers to scan oak populations in southwestern China and incorporating seed functional traits analyses are necessary to reveal the underlying drivers lead to the contemporary genetic structure found in these oaks.

### The Impacts of Ancient Geological Events on the Divergence of Subtropical Lineages in the Southeastern Himalaya Fringe

**FIGURE 7:**
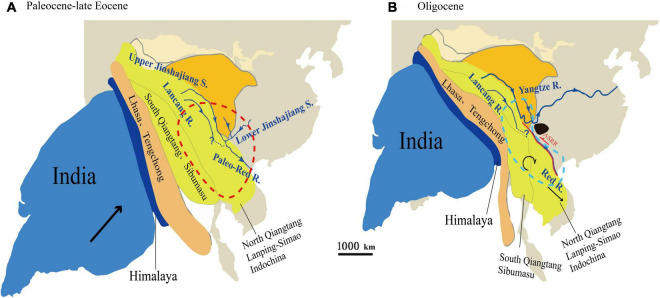
Schematic geodynamic evolutionary model of the paleo-drainage evolution of East Asia and schematic diagram of the ancestral distribution of the *Quercus franchetii* complex from the Eocene to Oligocene (based on Replumaz and Tapponnier, 2003; Royden et al., 2008; Yan and Chen, 2018). **(A)** Paleocene to Late Eocene. The red dotted area indicates the possible ancestral distribution area of the *Q. franchetii* complex in the Paleogene; **(B)** the Oligocene. The black area indicates the area where the early evolution of the ancient Red River was captured (Nanpan River area). The blue dotted area shows the ancient Red River area in the Oligocene period.

The Oligocene was a key period in the formation of the modern topography of China. During this period, the high eastern and low western topographies of China were totally reversed (Ming, 2007; Di et al., 2018). The tectonic plates associated with the spreading of the East Asian marginal sea and the uplift of the eastern margin of the Tibetan Plateau exerted important influence on the topography and drainage of East Asia (Clark et al., 2004). This was accompanied by dramatic tectonically induced topographic changes and landscape development, which resulted in repeated river captures and reversals (Zheng, 2015). Paleomagnetic and stratigraphic evidence suggests that there was a wide Paleo-Red River drainage basin between the southeastern Tibetan Plateau and the South China Sea, which included Hoh-Xil, Songpan-Ganzi, northern Qiangtang, Yidun, and western Yangtze Terranes (Figure 7). Since the late Eocene, the hard collision between the Indian and Eurasian plates began. These massifs/blocks were extruded eastward under the resulting pressure (Chen et al., 2017). Meanwhile, the Ailao Shan-Red River fault zone began to slip rapidly. The Lanpin-Simao block began to rotate clockwise substantially, which blocked the upper and lower Yangtze River from continually flowing to the south along the paleo-drainage (Figure 7). As a result, the northern drainage basin of the paleo Red River disappeared, and the modern Red River began to become established (Replumaz and Tapponnier, 2003; Royden et al., 2008; Chen et al., 2017; He et al., 2021). These the Oligocene to early Miocene events greatly changed the regional biota, e.g., giving rise to the diversification of *Cautleya*, *Roscoea* (Zhao et al., 2016), *Badidae* (Rüber et al., 2004), and spiny frogs (*Paini*) (Che et al., 2010).

Our ancestral range reconstruction based on *cp*DNA data showed that the *Q. franchetii* complex once had a wide distribution in southwestern China and the southern Himalaya regions during the early Oligocene, followed by three vicariance events. Among these events, the NPR (Clade I) was first diversified during the mid-Oligocene (ca. 30 Ma), and then, during the late Oligocene and the early Miocene, the RR lineage and HDM lineage were derived, respectively. Within the main lineages, the fast divergence of the *cp*DNA haplotypes occurred during the late Miocene (Figure 7A). The NPR was located at the core area during early river capture; the hard collision between the Indian and Eurasian plates might have squeezed the plates in this region leading to river re-alignment, which induced the divergence of the NPR lineage (Clark et al., 2004; Yang et al., 2012; Figure 7B). During the late Oligocene, the Ailao Shan-Red River fault had an early left strike-slip that may have raised the barrier that blocked gene flow among the populations and promoted allopatric divergence (Hall et al., 2008; Fyhn et al., 2009; Lin et al., 2009; Deng et al., 2014). Thus, the RR lineage diverged (Clade II). During the early Miocene, large fault basins were established corresponding to the fast uplift of the YGP and HDM regions (Zhang, 2012), which might dramatically change the regional topography and climate, eventually blocking gene flow in the two regions and leading to the divergence of the YGP lineage (Clade III) and HDM lineage (Clade IV). Followed by the late Neogene period fast HDM uplifts, the complex topography of southwestern China was eventually formed, which further restricted regional seed-mediated gene flow and promoted the divergence of *cp*DNA haplotypes. A similar scenario was also detected in other oaks, e.g., *Q. delavayi* (Xu et al., 2020), and *Q. aquifolioides* (Du et al., 2017), as well as plant lineages with wide distribution in semi-humid evergreen broadleaved forests on YGP region, e.g., *Primula secundiflora* (Wang et al., 2008), *Terminalia franchetii* (Zhang et al., 2011), and *Cycas multipinnata* (Gong et al., 2015). Such phenomenon suggested the regional biota might be impacted by similar environmental drivers.

In summary, the high biodiversity levels found in southwestern China are rooted deeply in the Oligocene. The early tectonic events during the Oligocene drove the main lineage splits, while the fast uplifts of the Himalayas during the Miocene-Pliocene increased environmental heterogeneity and established substantial dispersal barriers (Meng et al., 2017; Xing and Ree, 2017; Yuan et al., 2019). Then, the Quaternary climate fluctuation led to distribution range contractions and expansions of the species, as well as the occurrence of Asian winter monsoons, and the dry season in winter and spring in southwestern China further restricted gene flow between the core YGP region and its periphery (Su et al., 2013; Ye et al., 2019). All these geological and climatic factors interacted during different timespans to shape the contemporary divergence pattern of the biota of the southeastern Himalaya biodiversity hotspot. Our phylogeographic study indicated the Oligocene tectonic induced divergence in *Q. franchetii* complex, which is a supplement to the review of Renner (2016) concerning the Paleogene events contributing to the species richness of the East Himalayan biodiversity hotspot.

Moreover, the population genetic structures inferred from nSSR and *cp*DNA markers of *Q. franchet*ii complex were dissimilar. Notably, nSSRs mainly reflected the divergence between the populations in the core YGP region and the peripheral populations. The similar population genetic structure on nuclear genome was also reported in another sympatric species (*Q. cocciferoides*; Liu, 2019). All these evidences suggested the two species might underwent the similar selection pressure to trigger their divergence. The potential migration corridor analysis on the *Q. franchetii* complex showed that the populations in core YGP region maintained strong gene flows, but the marginal populations were mostly isolated since LGM (Figure 6). The rugged topography induced by the rapid YPG uplift during the late Neogene and (or) highly fragmented habitat of semi-humid evergreen forests in the peripheral areas around YGP might boost the allopatric divergence of these oaks. Nevertheless, another possibility of this pattern is that the quick evolution and possible backward evolution of SSR markers blurs the geological pattern. Further investigation using high throughput marker to illustrate the genetic structure at fine scale can provide a better understanding on the interplays between genetic diversity and environmental factors.

In contrast, the *cp*DNA data of *Q. franchetii* complex showed a much clearer phylogeographic structure, which has also been shown in other oaks, e.g., *Q. delavayi* (Xu et al., 2020), *Q. cocciferoides* (Liu, 2019), and *Q. aquifoliodes* (Du et al., 2017). Pollen- and seed-mediated gene flow have very different dispersal efficiencies in oaks (Du et al., 2017; Liu, 2019; Xu et al., 2020). In this study, nSSRs showed no significant differentiation of the populations in the East Red River, West Red River, and HDM regions, but *cp*DNA data showed a clear phylogeographic structure. This result suggested that these early tectonic activities during the Oligocene to early Miocene might have restricted seed-mediated gene flow in different regions, but only had limited impacts on pollen-induced gene flow.

In contrast, the niche modeling result suggested that the *Q. franchetii* complex populations are mainly located in the predicted suitable area. While the species complex experienced a southern contraction during the LGM, the distribution area at present and during the LGM largely overlapped in YGP. The quaternary glaciation had only minor impacts on its distribution. Likewise, the niche modeling results of *Osteomeles schwerinae* (Wang et al., 2015), *Q. schottkyana* (Jiang et al., 2016), and *Q. kerrii* (Jiang et al., 2018) from southwestern China show the similar pattern. Collectively, these studies suggest that central YGP region is an important refugia for species in semi-humid evergreen broadleaved forests in southwestern China.

## Conclusion and Perspective

The spatial genetic structure is subject to environmental factors and the evolutionary process of the organism that affect genetic and genomic variation. The southeastern Himalaya fringe with extensive environmental changes since Cenozic high biodiversity. In our case, the population genetic diversity pattern of the *Q. franchetii* complex showed that the divergence of this subtropical lineage is rooted at the Oligocene. The tectonic events ever since this epoch might have restricted the regional seed-mediated gene flow, in turn triggered the early divergences of this subtropical woody lineage (Replumaz and Tapponnier, 2003; Clark et al., 2004; Zhang et al., 2011). Following, the rapid uplift-induced environmental heterogeneity in the Miocene in the southeastern Himalayas fringe, with subsequent Quaternary climatic fluctuations inducing distribution range expansions and contractions might further restrict the gene flow among the populations in core distribution and the peripheral areas (Huchon et al., 1994; Liu et al., 2006; Xu et al., 2010). These geological and climatic factors acted in a combined manner to boost the diversification of the subtropical biota in the southeastern Himalaya fringe. Our study provides an example that clearly reveals the evolutionary dynamics of the subtropical evergreen forests since the Oligocene in southwestern China for the first time, and demonstrated that except for the biological traits, the evolutionary history of the lineages are important factors impact the spatial genetic structures found in the evergreen oaks in YGP region. These results can provide important information on the formation of high biodiversity level in southeast Himalayas, as well as conservation and safeguard this unique ecosystem on the background of global climate change.

## Data Availability Statement

The datasets presented in this study can be found in online repositories. The names of the repository/repositories and accession number(s) can be found below: https://www.ncbi.nlm. nih.gov/, MW201294-MW201314, MW201315-MW201334, and MW201335-MW201352.

## Author Contributions

MD and X-LJ conceived and designed the experiments and were responsible for field collections and specimen identification. S-SZ performed the experiments. S-SZ and X-LJ analyzed the data. MD, S-SZ, and X-LJ wrote and revised the manuscript. Q-JH gave much advice on the details of the manuscript. All authors contributed to the article and approved the submitted version.

## Conflict of Interest

The authors declare that the research was conducted in the absence of any commercial or financial relationships that could be construed as a potential conflict of interest.

## Publisher’s Note

All claims expressed in this article are solely those of the authors and do not necessarily represent those of their affiliated organizations, or those of the publisher, the editors and the reviewers. Any product that may be evaluated in this article, or claim that may be made by its manufacturer, is not guaranteed or endorsed by the publisher.

## Footnotes

1
http://www.esri.com/software/arcgis/

2
http://tree.bio.ed.ac.uk/software/tracer/

3
http://beast.bio.ed.ac.uk/TreeAnnotator

4
http://tree.bio.ed.ac.uk/software/figtree/

5
http://www.gbif.org

6
https://www.cvh.ac.cn

7
http://www.worldclim.org/

8
https://CRAN.R-project.org/package=dismo

9
https://chelsa-climate.org/

10
http://www.r-project.org/
