# Supplementary material for: Historical Dynamics of Semi-Humid Evergreen Forests in the Southeast Himalaya Biodiversity Hotspot: A Case Study of the Quercus franchetii Complex (Fagaceae)
Source: Front Plant Sci. 2021 Nov 23;12:774232. doi: 10.3389/fpls.2021.774232 (PMC8753985; doi:10.3389/fpls.2021.774232)

**ADDITIONAL FILES**

**APPENDIX S1.** Microsatellite and *cp*DNA used in population genetic structure scan of the *Quercus franchetii* complex.

|  | **Primer** | **Primer Sequence (5′ to 3′)** | **Annealing temperature (°C)** | **References** |
| --- | --- | --- | --- | --- |
| *cp*DNA sequences | *psb*A-*trnH* | GTTATGCATGAACGTAATGCTC  CGCGCATGGTGGATTCACAAATC | 55 | (Shaw et al., 2005) |
|  | *trn*T-*trn*L | CATTACAAATGCGATGCTCT  TCTACCGATTTCGCC ATA TC | 50 | (Taberlet et al., 1991) |
|  | *atp*I-*atp*H | ATAGGTGAATCCATGGAGGG  CCAGCAGCAATAACGGAAGC | 52 | (Grivet et al., 2005) |
|  | **Primer** | **Primer Sequence (5′ to 3′)** | **Repeat motif** | **References** |
| nSSR sequences | **QpZAG15** | (M13) CGATTTGATAATGACACTATGG  CATCGACTCATTGTTAAGCAC | (AG)_23_ | (Steinkellner et al., 1997) |
|  | **QrZAG112** | (M13) TTCTTGCTTTGGTGCGCG  GTGGTCAGAGACTCGGTAAGTATTC | (GA)_32_ | (Kampfer et al., 1998) |
|  | comp11050 | (M13) ATCCTACGCTTGTCGGGTC  TCGACAACCCACTCCTTGG | (AG)_7_ | (An et al., 2016) |
|  | **comp17611** | (M13) GCCTTGGAGGCGAAAGAAC  TCCTGGCTTCGGATCTTGG | (CTT)_5_ | (An et al., 2016) |
|  | comp19373 | (M13) AACCCGCCGATTCTCACAG  TCGAGGGCAGACTCGAATG | (AG)_6_ | (An et al., 2016) |
|  | bcqm42 | (M13) CAGTGGGACCTTCTTATGCC  GCTTTGGAACAACTGCTACATC | (GT)_11_ | (Mishima et al., 2006) |
|  | **RAD 191** | (M13) CAGAGCTGAGAACTGAGAA  AAACCCACTAGATTCCCAAA | (GA)_15_ | (Ning et al., 2020) |
|  | **RAD 213** | (M13) GCATAACCCACCTCTCAAT  CCAGAATCTATTACAACTCCG | (GGT)_9_ | (Ning et al., 2020) |

*Note*: M13 sequence: 5′-TGTAAAACGACGGCCAGT-3′. Primers in bold deviated from HWE (*P* < 0.05)

**APPENDIX S2.** Pairwise *F*_ST_-values between the studied localities of *Quercus franchetii complex*. Below diagonal were *F*_ST_-values based on SSR data and above were *F*_ST_-values based on cpDNA.

| Pop. | KZ | YS | EY | WD | ES | XP | YJ | SJ | LC | MD | JD | BC | LF | CH | GJ | ZS | TH | PE | DL | XS | YM | YL | HP | RH | PZH | NH | XC | MN | KY | QB | SL | XN | VM |
| --- | --- | --- | --- | --- | --- | --- | --- | --- | --- | --- | --- | --- | --- | --- | --- | --- | --- | --- | --- | --- | --- | --- | --- | --- | --- | --- | --- | --- | --- | --- | --- | --- | --- |
| KZ | - | 0.54 | 1 | 1 | 0.79 | 1 | 1 | 1 | 1 | 0.90 | 1 | 1 | 1 | 1 | 0.94 | 0.78 | 1 | 1 | 0.96 | 1 | 1 | 1 | 0.94 | 0.99 | 0.99 | 1 | 1 | 1 | 1 | 1 | 1 | 1 | 1 |
| YS | 0.18 | - | 0.87 | 0.54 | 0.48 | 0.89 | 0.89 | 0.63 | 0.63 | 0.55 | 0.54 | 0.54 | 0.54 | 0.09 | 0.74 | 0.49 | 0.58 | 0.69 | 0.63 | 0.67 | 0.64 | 0.86 | 0.85 | 0.86 | 0.85 | 0.86 | 0.94 | 0.89 | 0.93 | 0.73 | 0.73 | 0.54 | 0.93 |
| EY | 0.24 | 0.18 | - | 1 | 0.90 | 1 | 1 | 1 | 1 | 0.96 | 1 | 1 | 1 | 1 | 0.96 | 0.90 | 1 | 1 | 0.98 | 1 | 1 | 1 | 0.4 | 0.98 | 0.97 | 1 | 1 | 1 | 1 | 1 | 1 | 1 | 1 |
| WD | 0.15 | 0.06 | 0.16 | - | 0.73 | 1 | 1 | 1 | 1 | 0.87 | 1 | 1 | 1 | 1 | 0.93 | 0.74 | 1 | 1 | 0.95 | 1 | 1 | 1 | 0.94 | 0.98 | 0.99 | 1 | 1 | 1 | 1 | 1 | 1 | 1 | 1 |
| ES | 0.17 | 0.04 | 0.14 | -0.02 | - | 0.91 | 0.91 | 0.59 | 0.59 | 0.46 | 0.40 | 0.40 | 0.40 | -0.19 | 0.75 | 0.40 | 0.59 | 0.69 | 0.60 | 0.69 | 0.64 | 0.88 | 0.86 | 0.88 | 0.88 | 0.89 | 0.96 | 0.92 | 0.95 | 0.75 | 0.75 | 0.54 | 0.95 |
| XP | 0.19 | 0.10 | 0.1 | -0.03 | 0.06 | - | 0 | 1 | 1 | 0.96 | 1 | 1 | 1 | 1 | 0.97 | 0.91 | 1 | 1 | 0.98 | 1 | 1 | 1 | 0.92 | 0.97 | 0.99 | 1 | 1 | 1 | 1 | 1 | 1 | 1 | 1 |
| YJ | 0.21 | 0.14 | 0.26 | 0.08 | 0.13 | 0.14 | - | 1 | 1 | 0.96 | 1 | 1 | 1 | 1 | 0.97 | 0.91 | 1 | 1 | 0.98 | 1 | 1 | 1 | 0.92 | 0.97 | 0.99 | 1 | 1 | 1 | 1 | 1 | 1 | 1 | 1 |
| SJ | 0.23 | 0.18 | 0.21 | 0.14 | 0.14 | 0.01 | 0.19 | - | 0 | 0.11 | 1 | 1 | 1 | 1 | 0.91 | 0.58 | 1 | 1 | 0.91 | 1 | 1 | 1 | 0.92 | 0.98 | 0.98 | 1 | 1 | 1 | 1 | 1 | 1 | 1 | 1 |
| LC | 0.15 | 0.15 | 0.22 | -0.02 | 0.08 | 0.06 | 0.13 | 0.08 | - | 0.11 | 1 | 1 | 1 | 1 | 0.91 | 0.58 | 1 | 1 | 0.91 | 1 | 1 | 1 | 0.92 | 0.98 | 0.98 | 1 | 1 | 1 | 1 | 1 | 1 | 1 | 1 |
| MD | 0.15 | 0.25 | 0.29 | 0.09 | 0.18 | 0.21 | 0.29 | 0.26 | 0.14 | - | 0.56 | 0.56 | 0.56 | 0.11 | 0.83 | 0.45 | 0.76 | 0.83 | 0.74 | 0.84 | 0.74 | 0.95 | 0.89 | 0.94 | 0.94 | 0.95 | 0.98 | 0.96 | 0.98 | 0.87 | 0.87 | 0.74 | 0.98 |
| JD | 0.22 | 0.23 | 0.26 | 0.03 | 0.16 | 0.21 | 0.24 | 0.26 | 0.16 | 0.18 | - | 0 | 0 | 0 | 0.89 | 0.39 | 1 | 1 | 0.86 | 1 | 1 | 1 | 0.93 | 0.98 | 0.98 | 1 | 1 | 1 | 1 | 1 | 1 | 1 | 1 |
| BC | 0.12 | 0.27 | 0.28 | 0.09 | 0.21 | 0.23 | 0.26 | 0.27 | 0.16 | 0.05 | 0.16 | - | 0 | 0 | 0.89 | 0.39 | 1 | 1 | 0.86 | 1 | 1 | 1 | 0.93 | 0.98 | 0.98 | 1 | 1 | 1 | 1 | 1 | 1 | 1 | 1 |
| LF | 0.07 | 0.22 | 0.24 | 0.09 | 0.18 | 0.21 | 0.22 | 0.24 | 0.13 | 0.10 | 0.14 | 0.04 | - | 0 | 0.89 | 0.39 | 1 | 1 | 0.86 | 1 | 1 | 1 | 0.93 | 0.98 | 0.98 | 1 | 1 | 1 | 1 | 1 | 1 | 1 | 1 |
| CH | 0.15 | 0.24 | 0.20 | 0.01 | 0.17 | 0.16 | 0.22 | 0.23 | 0.12 | 0.04 | 0.12 | 0.03 | 0.04 | - | 0.78 | -0.2 | 1 | 1 | 0.73 | 1 | 1 | 1 | 0.85 | 0.96 | 0.96 | 1 | 1 | 1 | 1 | 1 | 1 | 1 | 1 |
| GJ | 0.22 | 0.19 | 0.19 | 0.11 | 0.14 | 0.18 | 0.19 | 0.22 | 0.18 | 0.23 | 0.25 | 0.27 | 0.22 | 0.21 | - | 0.75 | 0.91 | 0.92 | 0.89 | 0.91 | 0.90 | 0.96 | 0.92 | 0.95 | 0.95 | 0.96 | 0.98 | 0.9 | 0.98 | 0.93 | 0.93 | 0.87 | 0.98 |
| ZS | 0.16 | 0.13 | 0.23 | 0.04 | 0.06 | 0.20 | 0.19 | 0.22 | 0.13 | 0.23 | 0.22 | 0.23 | 0.15 | 0.22 | 0.14 | - | 0.58 | 0.68 | 0.60 | 0.69 | 0.64 | 0.89 | 0.86 | 0.89 | 0.88 | 0.89 | 0.96 | 0.92 | 0.95 | 0.74 | 0.74 | 0.54 | 0.95 |
| TH | 0.16 | 0.08 | 0.18 | -0.01 | 0.02 | 0.05 | 0.06 | 0.12 | 0.04 | 0.19 | 0.20 | 0.22 | 0.19 | 0.17 | 0.13 | 0.08 | - | 1 | 0.91 | 1 | 1 | 1 | 0.93 | 0.98 | 0.98 | 1 | 1 | 1 | 1 | 1 | 1 | 1 | 1 |
| PE | 0.35 | 0.34 | 0.38 | 0.35 | 0.35 | 0.35 | 0.41 | 0.41 | 0.39 | 0.38 | 0.36 | 0.37 | 0.35 | 0.36 | 0.39 | 0.42 | 0.39 | - | 0.94 | 1 | 1 | 1 | 0.93 | 0.98 | 0.9 | 1 | 1 | 1 | 1 | 1 | 1 | 1 | 1 |
| DL | 0.18 | 0.12 | 0.24 | 0.02 | 0.10 | 0.17 | 0.21 | 0.21 | 0.13 | 0.15 | 0.22 | 0.21 | 0.18 | 0.18 | 0.14 | 0.14 | 0.13 | 0.34 | - | 0.94 | 0.93 | 0.98 | 0.92 | 0.97 | 0.96 | 0.98 | 0.99 | 0.99 | 0.99 | 0.95 | 0.95 | 0.89 | 0.99 |
| XS | 0.20 | 0.08 | 0.23 | -0.003 | 0.06 | 0.07 | 0.18 | 0.15 | 0.09 | 0.18 | 0.22 | 0.25 | 0.21 | 0.20 | 0.14 | 0.13 | 0.08 | 0.35 | 0.03 | - | 1 | 1 | 0.92 | 0.98 | 0.98 | 1 | 1 | 1 | 1 | 1 | 1 | 1 | 1 |
| YM | 0.20 | 0.19 | 0.19 | 0.23 | 0.25 | 0.20 | 0.29 | 0.27 | 0.27 | 0.26 | 0.24 | 0.23 | 0.21 | 0.23 | 0.32 | 0.32 | 0.28 | 0.30 | 0.26 | 0.27 | - | 1 | 0.92 | 0.98 | 0.98 | 1 | 1 | 1 | 1 | 1 | 1 | 1 | 1 |
| YL | 0.30 | 0.28 | 0.24 | 0.30 | 0.28 | 0.28 | 0.36 | 0.31 | 0.33 | 0.35 | 0.29 | 0.30 | 0.29 | 0.31 | 0.36 | 0.35 | 0.33 | 0.41 | 0.30 | 0.34 | 0.11 | - | 0.88 | 0.94 | 0.99 | 1 | 1 | 1 | 1 | 1 | 1 | 1 | 1 |
| HP | 0.26 | 0.26 | 0.26 | 0.29 | 0.32 | 0.28 | 0.36 | 0.35 | 0.34 | 0.30 | 0.31 | 0.27 | 0.27 | 0.25 | 0.38 | 0.41 | 0.35 | 0.33 | 0.30 | 0.33 | 0.03 | 0.23 | - | 0.88 | 0.92 | 0.93 | 0.97 | 0.94 | 0.96 | 0.94 | 0.94 | 0.90 | 0.96 |
| RH | 0.24 | 0.26 | 0.27 | 0.35 | 0.32 | 0.29 | 0.37 | 0.35 | 0.33 | 0.31 | 0.33 | 0.28 | 0.24 | 0.26 | 0.38 | 0.38 | 0.35 | 0.35 | 0.30 | 0.33 | 0.03 | 0.22 | 0.03 | - | 0.97 | 0.98 | 0.99 | 0.99 | 0.99 | 0.98 | 0.98 | 0.97 | 0.99 |
| PZH | 0.27 | 0.26 | 0.26 | 0.29 | 0.31 | 0.28 | 0.38 | 0.34 | 0.31 | 0.28 | 0.31 | 0.26 | 0.25 | 0.23 | 0.38 | 0.40 | 0.35 | 0.35 | 0.29 | 0.32 | 0.05 | 0.21 | 0.02 | 0.01 | - | 0.97 | 0.99 | 0.99 | 0.99 | 0.98 | 0.98 | 0.98 | 0.99 |
| NH | 0.11 | 0.22 | 0.24 | 0.12 | 0.22 | 0.17 | 0.26 | 0.21 | 0.17 | 0.15 | 0.19 | 0.14 | 0.10 | 0.11 | 0.26 | 0.25 | 0.22 | 0.34 | 0.17 | 0.2 | 0.17 | 0.28 | 0.22 | 0.21 | 0.21 | - | 1 | 1 | 1 | 1 | 1 | 1 | 1 |
| XC | 0.25 | 0.29 | 0.28 | 0.32 | 0.34 | 0.29 | 0.38 | 0.37 | 0.34 | 0.27 | 0.33 | 0.25 | 0.27 | 0.24 | 0.39 | 0.42 | 0.37 | 0.33 | 0.30 | 0.32 | 0.07 | 0.26 | 0.01 | 0.05 | 0.05 | 0.20 | - | 1 | 1 | 1 | 1 | 1 | 1 |
| MN | 0.26 | 0.27 | 0.24 | 0.30 | 0.31 | 0.26 | 0.38 | 0.36 | 0.34 | 0.27 | 0.32 | 0.25 | 0.28 | 0.25 | 0.37 | 0.40 | 0.35 | 0.31 | 0.30 | 0.31 | 0.06 | 0.23 | 0.03 | 0.08 | 0.08 | 0.23 | 0.01 | - | 1 | 1 | 1 | 1 | 1 |
| KY | 0.24 | 0.23 | 0.22 | 0.19 | 0.22 | 0.24 | 0.32 | 0.31 | 0.26 | 0.23 | 0.17 | 0.21 | 0.19 | 0.211 | 0.30 | 0.30 | 0.29 | 0.30 | 0.28 | 0.28 | 0.10 | 0.22 | 0.13 | 0.16 | 0.14 | 0.24 | 0.16 | 0.12 | - | 1 | 1 | 1 | 1 |
| QB | 0.19 | 0.21 | 0.19 | 0.22 | 0.23 | 0.25 | 0.32 | 0.29 | 0.25 | 0.23 | 0.25 | 0.19 | 0.16 | 0.20 | 0.29 | 0.28 | 0.28 | 0.31 | 0.25 | 0.28 | 0.05 | 0.16 | 0.12 | 0.07 | 0.07 | 0.18 | 0.14 | 0.13 | 0.07 | - | 0 | 1 | 1 |
| SL | 0.27 | 0.25 | 0.21 | 0.24 | 0.25 | 0.28 | 0.36 | 0.32 | 0.28 | 0.28 | 0.27 | 0.25 | 0.21 | 0.24 | 0.35 | 0.33 | 0.31 | 0.35 | 0.26 | 0.31 | 0.09 | 0.16 | 0.15 | 0.13 | 0.09 | 0.23 | 0.18 | 0.15 | 0.12 | 0.02 | - | 1 | 1 |
| XN | 0.24 | 0.24 | 0.31 | 0.19 | 0.22 | 0.28 | 0.25 | 0.30 | 0.21 | 0.25 | 0.12 | 0.21 | 0.16 | 0.18 | 0.31 | 0.28 | 0.26 | 0.43 | 0.26 | 0.23 | 0.28 | 0.39 | 0.34 | 0.35 | 0.35 | 0.22 | 0.36 | 0.37 | 0.20 | 0.28 | 0.35 | - | 1 |
| VM | 0.32 | 0.29 | 0.30 | 0.25 | 0.28 | 0.28 | 0.37 | 0.36 | 0.33 | 0.34 | 0.30 | 0.36 | 0.34 | 0.32 | 0.33 | 0.35 | 0.33 | 0.22 | 0.31 | 0.32 | 0.29 | 0.33 | 0.34 | 0.35 | 0.35 | 0.33 | 0.35 | 0.32 | 0.27 | 0.30 | 0.35 | 0.37 | - |

**APPENDIX S3.** The correlation between the genetic and geographic distance based on (**A**) *cp*DNA data and (**B**) nSSR data. The genetic distances of *cp*DNA and nSSRs data expressed as *F*_ST_ and *F*_ST_ (1 - *F*_ST_), respectively.


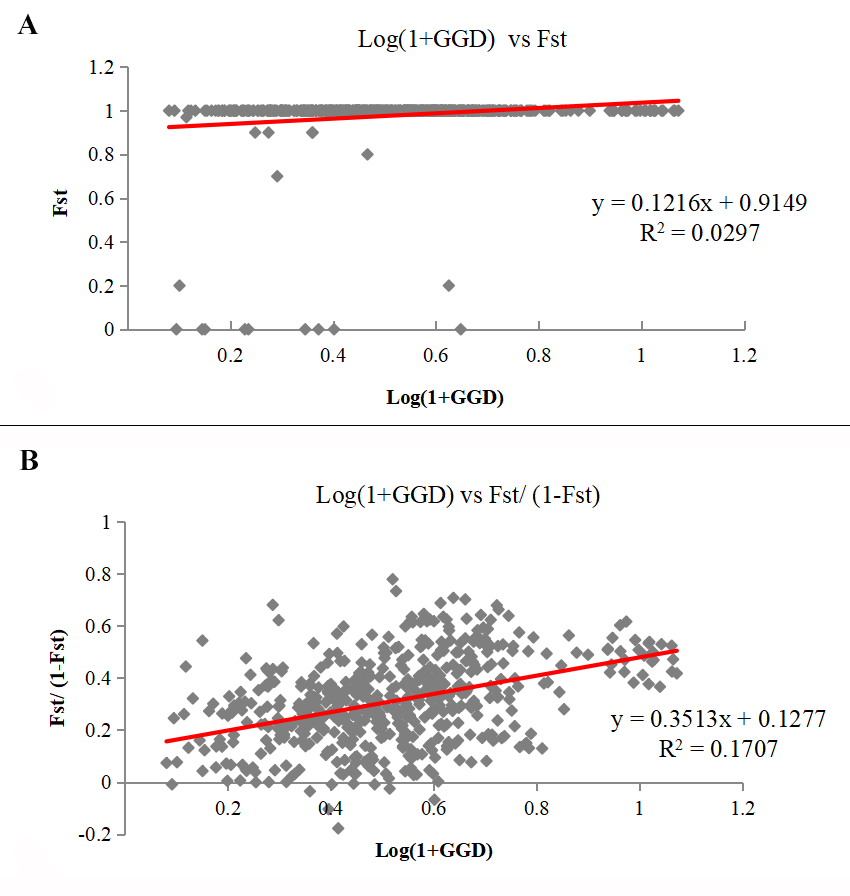


**APPENDIX S4.** Distribution of delta *K* values in (A) InStruct and (B) Structure analysis.


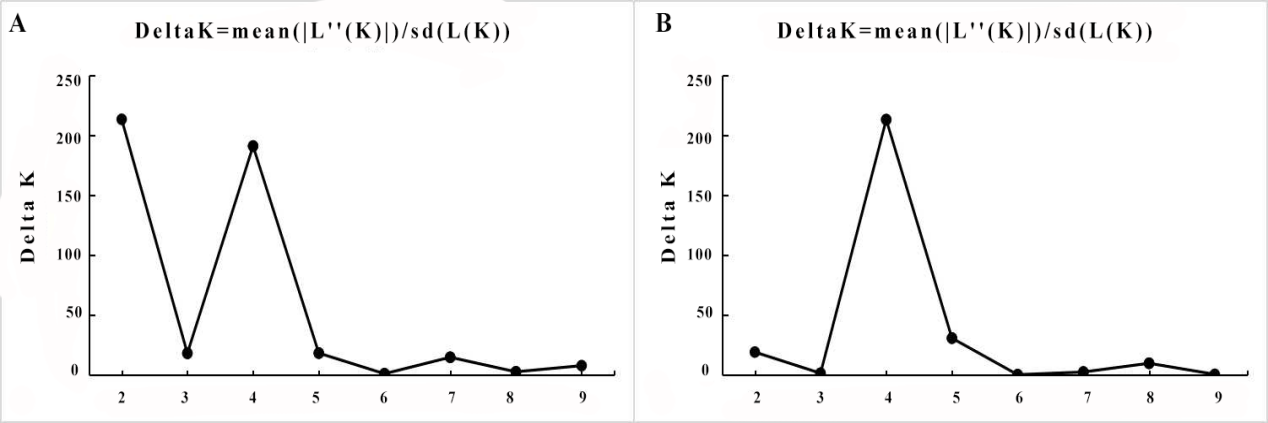


**APPENDIX S5.** (A) Geographic distribution of the *Quercus franchetii* complex according to the STRUCTURE grouping analysis. STRUCTURE cluster analysis diagram when (B) *K* = 2 and (C) *K* = 4. The colors in the pie charts represent the different groupings.


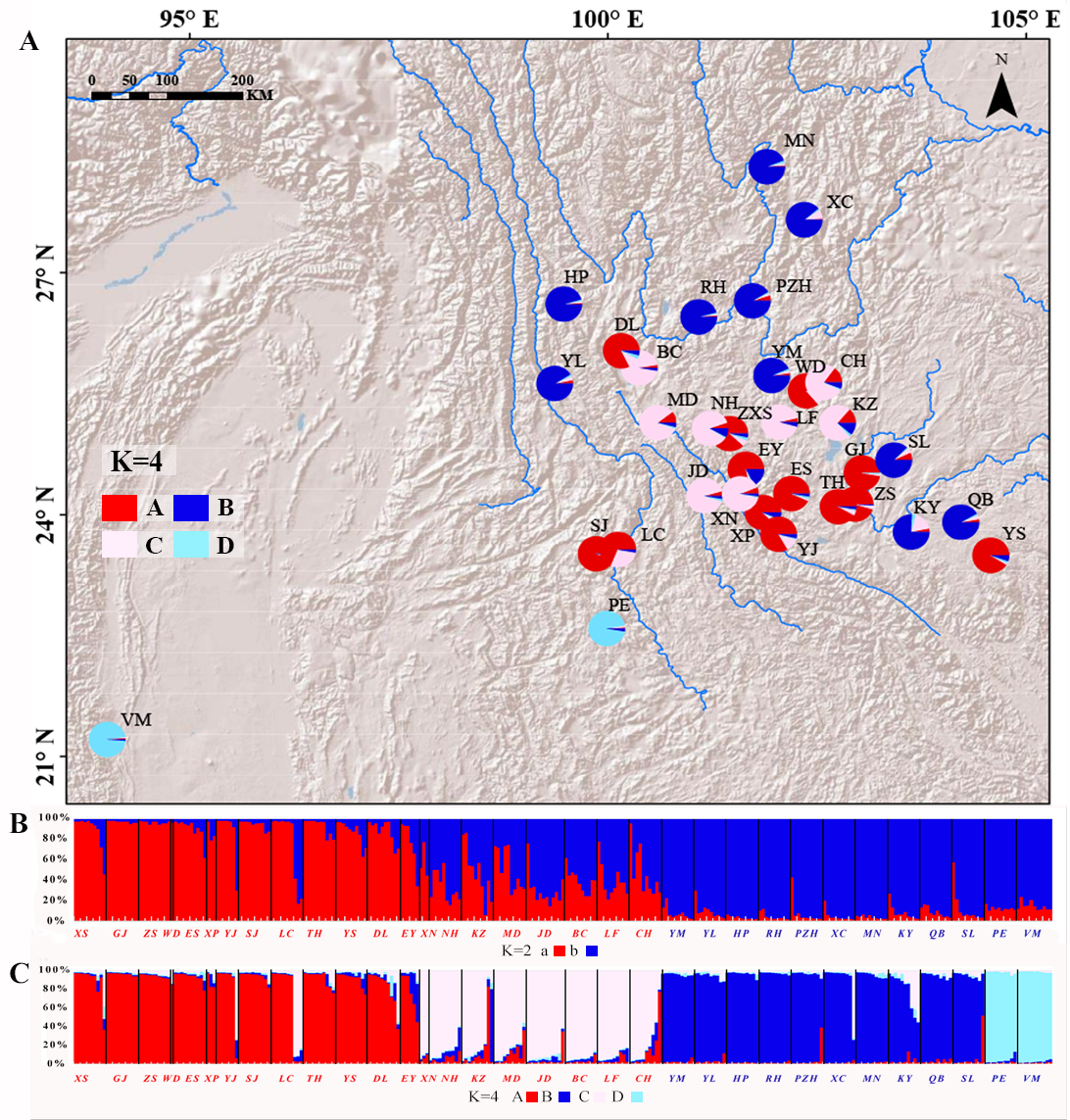


**APPENDIX S6.** Population structure of the *Quercus franchetii* complex based on nSSRs as inferred by principal coordinate analysis (PCoA).


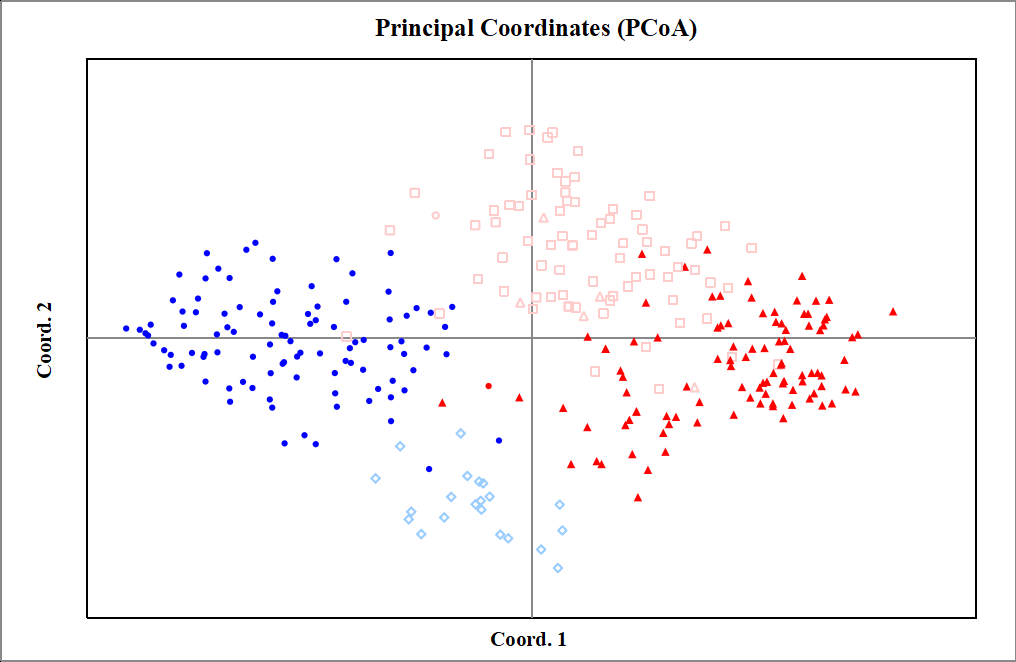


**APPENDIX S7.** Mismatch distribution for populations of the *Quercus franchetii* complex. The black bar and red dashed line represent the observed and expected mismatch distributions.


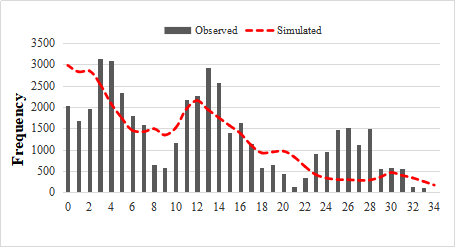


**APPENDIX S8.** Ancestral area reconstructions based on the Dispersal-Extinction-Cladogenesis (DEC) method implemented in RASP using the BEAST-derived chronogram of the *Quercus franchetii* complex. A, Nanpan River region (NPR); B, Southern Himalayas-southwestern Red River (RR); C, Hengduan Mountains area (HDM); D, Yunnan–Guizhou Plateau (YGP).


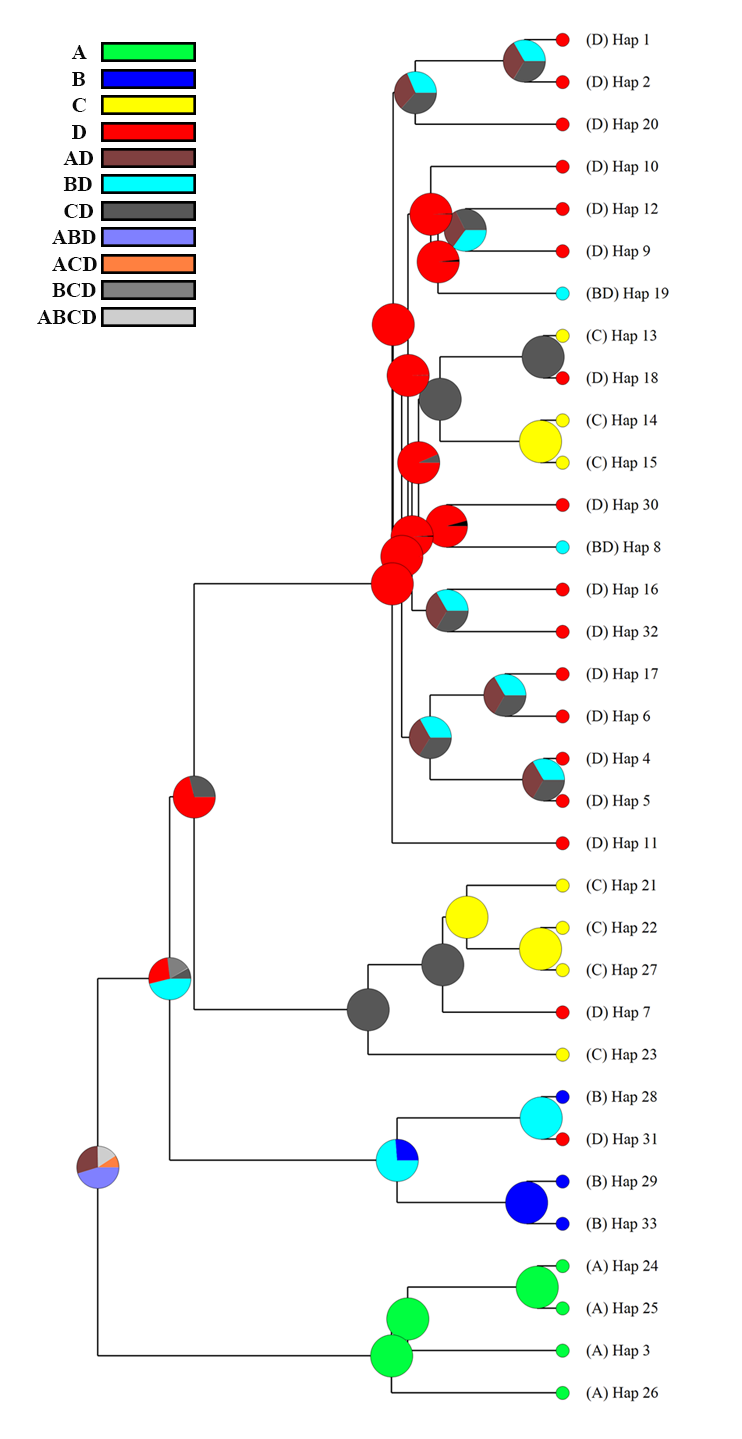


**APPENDIX S9.** Simulated distribution range of the *Quercus franchetii* complex during the present period using WorldClim and CHELSA databases. The red region showed the overlapped predicted present distribution range using WorldClim and CHELSA databases for simulation. The purple and the yellow represent the predicted distribution only obtained from WorldClim and CHELSA databases, respectively.


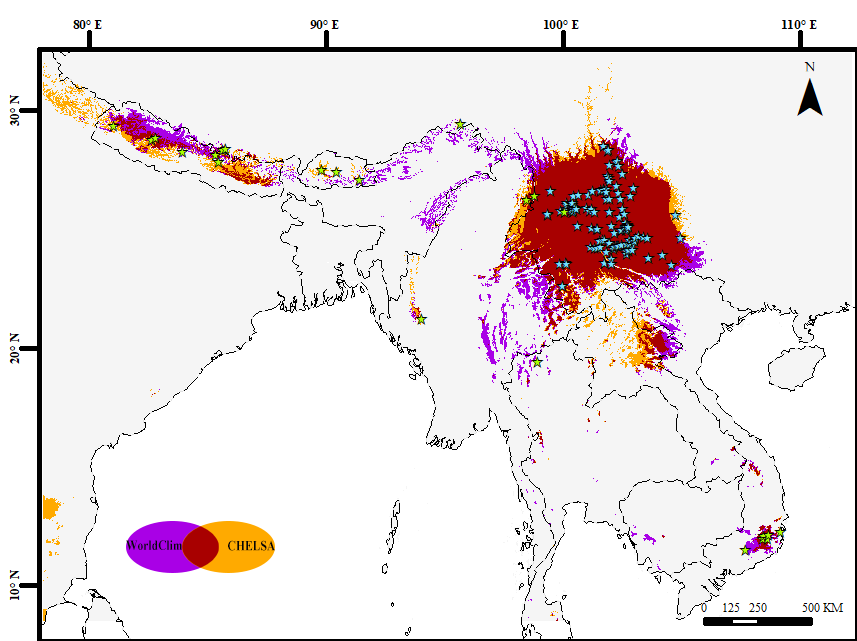

Supplement: Supplementary file 1 [file Data_Sheet_1.docx]
